# Epigenetic modifications in hyperhomocysteinemia: potential role in diabetic retinopathy and age-related macular degeneration

**DOI:** 10.18632/oncotarget.24333

**Published:** 2018-01-29

**Authors:** Khaled Elmasry, Riyaz Mohamed, Isha Sharma, Nehal M. Elsherbiny, Yutao Liu, Mohamed Al-Shabrawey, Amany Tawfik

**Affiliations:** ^1^ Department of Oral Biology and Anatomy, Dental College of Georgia, Augusta University, Augusta, GA, USA; ^2^ Department of Cellular Biology and Anatomy, Medical College of Georgia (MCG), Augusta University, Augusta, GA, USA; ^3^ James and Jean Culver Vision Discovery Institute, MCG, Augusta University, August, GA, USA; ^4^ Department of Human Anatomy and Embryology, Faculty of Medicine, Mansoura University, Mansoura, Egypt; ^5^ Department of Biochemistry, Faculty of Pharmacy, Mansoura University, Mansoura, Egypt

**Keywords:** epigenetic modifications, blood retinal barrier, diabetic retinopathy, age-related macular degeneration, hyperhomocysteinemia, Gerotarget

## Abstract

To study Hyperhomocysteinemia (HHcy)-induced epigenetic modifications as potential mechanisms of blood retinal barrier (BRB) dysfunction, retinas isolated from three- week-old mice with elevated level of Homocysteine (Hcy) due to lack of the enzyme cystathionine β-synthase (*cbs^–/–^*, *cbs^+/–^* and *cbs^+/+^*), human retinal endothelial cells (HRECs), and human retinal pigmented epithelial cells (ARPE-19) treated with or without Hcy were evaluated for (1) histone deacetylases (HDAC), (2) DNA methylation (DNMT), and (3) miRNA analysis. Differentially expressed miRNAs in mice with HHcy were further compared with miRNA analysis of diabetic mice retinas (STZ) and miRNAs within the exosomes released from Hcy-treated RPEs. Differentially expressed miRNAs were further evaluated for predicted target genes and associated pathways using Ingenuity Pathway Analysis. HHcy significantly increased HDAC and DNMT activity in HRECs, ARPE-19, and *cbs* mice retinas, whereas inhibition of HDAC and DNMT decreased Hcy-induced BRB dysfunction. MiRNA profiling detected 127 miRNAs in *cbs^+/–^* and 39 miRNAs in *cbs^–/–^* mice retinas, which were significantly differentially expressed compared to *cbs^+/+^*. MiRNA pathway analysis showed their involvement in HDAC and DNMT activation, endoplasmic reticulum (ER), and oxidative stresses, inflammation, hypoxia, and angiogenesis pathways. Hcy-induced epigenetic modifications may be involved in retinopathies associated with HHcy, such as age-related macular degeneration and diabetic retinopathy.

## INTRODUCTION

Homocysteine (Hcy) is an amino acid containing a sulfur group and is a key component of the methionine recycle system. Hcy plays a fundamental role in methyl-donor biosynthesis, which is biochemically linked to the principal epigenetic code found in DNA [1]. Our previous studies have reported that elevated Hcy, known as hyperhomocysteinemia (HHcy), disrupts inner [2, 3] and outer [4] blood-retinal barrier (BRB) integrity. The presence of an intact BRB is critical for maintaining the structural and functional integrity of the retina. The breakdown of BRB is a hallmark of the pathogenesis and development of retinal diseases, such as diabetic retinopathy (DR) [5–7] or age-related macular degeneration (AMD) [8–10]. Over the last decade, HHcy has received special attention to its relation to DR and AMD in several clinical and epidemiological studies, suggesting an association between elevated levels of serum Hcy and the risk of DR [11–16] and AMD [17–22]. However, the underlying mechanism of HHcy-induced BRB dysfunction is not clearly defined.

Mounting evidence revealed that elevated Hcy may be associated with DNA methylation and other epigenetic modifications [23–30]. Exploring the interactions among epigenome, genome, and environmental changes will increase the understanding of epigenetic-disease mechanisms and could lead to recognizing disease-risk factors in support of targeted intervention and therapies [31–34].

Epigenetic modifications are defined as changes in phenotype and gene expression that take place without alterations of DNA sequence [35]. Recently, epigenetic modifications have been highlighted as important mechanisms for the development of number of diseases [36–38]. There are three major epigenetic modification mechanisms:(1) DNA methylation, which is the most well-known epigenetic modification and plays an essential role in controlling global and specific gene expression. It is mediated by DNA methyl transferases (DNMTs) [39, 40], (2) Histone modification, which is defined as changes occurring in histone proteins that will affect gene expression.

Chromatin is the complex of chromosomal DNA and proteins that forms chromosomes in the nucleus. DNA is packaged in chromatin by wrapping around histone proteins in units called nucleosomes. Histone proteins are highly alkaline, with Histone tails that are normally positively charged due to presence of amine groups on their basic lysine and arginine amino acids [41, 42]. These positive charges on histone tails help histone proteins to interact with and bind to the DNA backbone at the negatively charged phosphate groups. During the acetylation process, amines on the histone change into amides; thereby, positive charges on the histone become neutralized and subsequently the capability of the histones to bind with DNA is reduced [43, 44]. This decreased binding results in expansion of chromatin, which permits genetic transcription to occur. The Histone deacetylation is catalyzed when histone deacetylases remove those acetyl groups, which increase the positively charged amine groups of histone tails and facilitate high-affinity binding between the histones and the negatively charged phosphate groups on the DNA backbone. The increased binding of DNA to histone condenses the DNA structure, interfering with the DNA transcription process [45].

Lastly, (3) non-coding RNA regulation (miRNAs), or microRNAs, comprise a unique class of small (~22) groups of nucleotides, endogenous non-coding RNAs that play a crucial role in regulating gene expression by silencing their target mRNAs [46–48]. MiRNAs can represent tools for the regulation of gene expression not only within the cell in which they are produced but also in the gene expression of other remote cells [49–51]. MiRNAs can be released from their cells of origin and circulate to target different cells in different tissues. One of the most interesting carriers of miRNAs are extracellular vesicles.

Extracellular vesicles, including exosomes, are nano-sized (30–150 nm) vesicles that are normally released into different body fluids to serve as an important intercellular communication tool [49, 50]. Exosomes have been reported to be related to different disease pathologies, including DR [51–55] and AMD [56–59]. The results of many earlier studies have suggested that exosomes play an important role in the pathogenesis of AMD and the contribution of retinal pigment epithelium (RPE)-derived exosomes to drusen formation [59–61], transport of oxidative stress signaling molecules from RPE to other retinal cells [56], and choroidal neovascularization [58]. Interestingly, circulating exosomal miRNAs have been studied as possible markers for AMD [62], and RPE-exosomes have been suggested as biological biomarkers in patients with neovascular AMD [57].

This study explores epigenetic modifications as possible mechanisms of HHcy-induced BRB dysfunction. Targeting HHcy-induced epigenetic modifications could provide therapeutic targets to mitigate Hcy-mediated BRB dysfunction in retinal diseases associated with elevated Hcy, such as DR and AMD.

## METHODOLOGY

### Cell culture

Human primary retinal endothelial cells (HRECs) were purchased from Cell Systems Cooperation (Kirkland, WA) and grown on plates coated with gelatin in EBM2 Medium (Catalog #190860, Lonza, Walkersville, MD) supplemented with 5% fetal bovine serum (FBS) and1% penicillin streptomycin (PS, Catalog # 30-004-CI, Corning, Inc., NY).

Human retinal pigmented epithelial cell line (ARPE-19) was attained from American Type Culture Collection (ATCC, Manassas, VA, USA). Passages 6–15 of ARPE-19 were grown on gelatin-coated dishes and incubated in Dulbecco's modified Eagle's medium-nutrient mixture F-12 (DMEM/F-12, Thermo Scientific, Wyman, MA) supplemented with 1% penicillin/streptomycin and 10% FBS (Atlantic Biological, Norcross, GA, USA). When the cells reached 80–90% confluency, they were shifted to the serum starvation overnight, followed by treatment with Hcy (20, 50 and 100 μM) or vehicle.

### Animal studies

Animal protocol was approved by the Institutional Animal Care and Use Committee (IACUC) at Augusta University. All experimental procedures done with animals were performed according to guidelines accepted by the Association for Research in Vision and Ophthalmology (ARVO) Statement for the Use of Animals in Ophthalmic and Vision Research. Cystathionine beta-synthase (*cbs*) deficient mice were generated as described before [63]. Colonies of *cbs^+/+^, cbs^+/−^,* and *cbs^−/−^* were established by breeding pairs of *cbs^+/−^* mice (B6.129P2-Cbstm1Unc/J; Jackson Laboratories, Bar Harbor, ME).

### Assessment of DNA methyltransferase (DNMT) activity

Nuclear extracts were prepared from retinas of *cbs^+/+^*, *cbs^+/–^* and *cbs^–/–^* mice (3 weeks old), HRECs and ARPE-19 treated with Hcy (20, 50 and 100 μM) using a nuclear extraction kit purchased from Abcam (Abcam Inc., Cambridge, MA, USA). Following extraction DNMT activity was assayed calorimetrically according to manufacturer's instructions (Cat# P-3009-48, Epigentek, Farmingdale, NY, USA). Briefly, nuclear extracts were added to a microplate coated with a universal DNMT substrate in presence of adenosyl methionine as methyl donor. The produced methylated DNA which is proportional to the DNMT activity was captured using anti-methyl cytosine antibody and then detected using detection antibody through an ELISA-like reaction. The color produced was measured at 450 nm and its density was proportional to DNMT activity.

### Assessment of HDAC activity

HDAC activity was assayed by using a colorimetric assay kit (Catalog #K331-100, BioVision, CA, USA). Retinal nuclear extracts prepared from *cbs^+/+^*, *cbs^+/–^* and *cbs^–/–^* mice and Hcy-treated HRECs and ARPE-19 (20, 50 and 100 μM) were incubated with a substrate containing acetylated Lysine side chain for 1 h at 37°C. Following deacetylation by sample HDAC, the reaction was terminated by adding Lysine Developer to produce a chromophore that was measured spectrophotometrically at 400 nm.

### Optical coherence tomography (OCT) and fluorescein angiography (FA)

To evaluate the effect of combined Inhibition of DNA methylation and histone deacetylation on Hcy-induced retinal disruption on 24-week-old mice injected intravitreally with Hcy with and without repeated intraperitoneal injection of combined inhibitors of DNA methylation and histone deacetylation. OCT and FA were performed simultaneously after 48 and 72 hours from Hcy injection, as described in our pervious publication [2, 4]. Briefly, mice were injected intravitreally with Hcy (200 μM) with and without intraperitoneal injection of combined inhibitor of HDAC, Sodium butyrate (SB, 1mg/kg, catalog # B5887-250MG sigma Aldrich) and DNMT inhibitor, 5-Azacytidine (5-AZC,2.5 mg/kg, catalog # A2385 SIGMA) [64–67]. The mice were anesthetized using 2% isoflurane and their eyes were dilated using 1% tropicamide eye drop. Each mouse was then placed on the imaging platform of the Phoenix Micron III retinal imaging microscope supplemented with OCT imaging device (Phoenix Research Laboratories, Pleasanton, CA). To keep the eye moist during imaging, lubricant gel was applied 20 μL 10% fluorescein sodium (Apollo Ophthalmics, Newport Beach, CA) were injected into the mice intraperitoneal, followed by rapid acquisition of fluorescent images ensued for ~5 minutes. Fluorescein leakage was demonstrated as indistinct vascular borders progressing to diffusely hazy fluorescence.

### RNA isolation, preparation and analysis

Retinas of age matched *cbs^+/–^*, *cbs^–/–^* and WT mice were enucleated and total RNA was extracted using Ambion, TRIzol Reagent (Life Technologies). RNA purity and concentration were assessed by spectrophotometry using Nano Drop ND-1000 (Thermo Fisher).

### Microarray analysis

A total 250 ng of RNA was labeled with biotin using the Flash Tag Biotin HSR RNA Labeling Kit (Affymetrix, Santa Clara, CA) according to the manufacturer's protocol. The labeled samples were then hybridized into the Gene Chip miRNA 3.0 array (Affymetrix). Hybridization, washing, and scanning of the arrays were carried out according to Affymetrix's recommendations. The data was imported into the Partek Genomic Suites version 6.6 (Partek, St. Louis, MO). Principal component analysis (PCA) was performed to visualize the partition among the groups. The differential expressions were calculated by using ANOVA of the Partek Package and filtered with a *p*-value cutoff of 0.05 and a fold-change cutoff 1.5-foldto screen highly significant miRNAs. The significant miRNA lists were used to generate hierarchical clustering plots.

### Bioinformatics analysis of the data

MiRNA lists were imported into Ingenuity Pathway Analysis (Qiagen) and analyzed on MicroRNA Target Filter and Core Analysis for detection of predicted target genes of miRNAs and associated pathways.

### RT-PCR

RT-PCR was performed to validate the differential expression of selected miRNAs. RNA was extracted from retinas of *cbs^+/–^* mice and their age matched controls. RT-PCR was performed for miRNAs in these samples using appropriate miScript primers obtained from Qiagen. The following primers were used: Mm_miR-200c_1 miScript Primer Assay (MS00001827), Mm_miR-205_1 miScript Primer Assay (MS00001862), Mm_miR-199a-3p_1 miScript Primer Assay (MS00007889), Mm_miR-206_1 miScript Primer Assay (MS00001869), Mm_miR-31_1 miScript Primer Assay (MS00001407), Mm_miR-16_2 miScript Primer Assay (MS00037366), Mm_miR-27b_1 miScript Primer Assay (MS00001358), Mm_miR-29a_1 miScript Primer Assay (MS00001372). U6 was used as an internal control (U6 snRNA, Product #.:203907, EXIQON)

### Exosmoes isolation from cell culture media

Conditional culture media was collected, and then centrifuged for 30 minutes at 2000 × g to remove any cells or debris. Then, exosomes were isolated using Invitrogen Total Exosome Isolation Reagent (from cell culture media) (Catalog#: 4478359) according to the manufacturer's protocol. Briefly, 0.5 volumes of the exosome isolation reagent was added to the media then vortexing was done to mix the reagent with the media. The mixture was left overnight at 4°C. Next day, the mixture was centrifuged at 10,000 × g for an hour at 4°C. Supernatants were aspirated and pellets were suspended again in an appropriate PBS volume.

### Zeta view nanoparticle tracking analysis (NTA)

The size and concentration of the exosomes isolated were quantified by using NTA which was done utilizing the ZetaView PMX 110 (Particle Metrix, Meerbusch, Germany) and its related software (ZetaView 8.02.28) [68]. Each sample was measured at 11 different positions, and then the size and concentration of each sample were obtained. ZetaView 8.02.28 software was used for the analysis of measurement data from the ZetaView.

### Exosomal RNA isolation and measurement

RNA isolation was performed using the Total Exosome RNA & Protein Isolation Kit (catalog # 4478545; Invitrogen) according to the manufacturer's protocol. A final volume of 30 μl RNA solution was collected from each. Agilent 2100 Bioanalyzer (Santa Clara, CA) was used for measuring RNA quality and concentration at the Integrated Genomics Core of Georgia Cancer Center at Augusta University.

### Statistical analysis

Different experimental groups were compared by using the student two-tailed *t* test or one-way analysis of variance (ANOVA), with a post hoc Tukey's test. Data considered statistically significant when *P* value < 0.05. The outcomes are expressed as mean ± SD. For the microarray data analysis; the fold change was calculated using least squares means of the groups.

## RESULTS

### Hcy increases DNA methylation and histone deacetylation *in vivo* and *in vitro*

**Figure 1:**
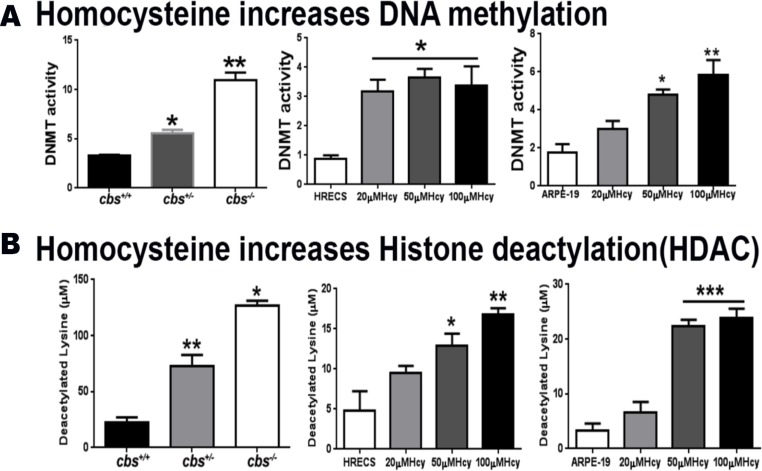
Assessment of DNA methylation and histone deacetylation (**A**) Measurement of DNA methyl transferase Enzyme (DNMT) activity in *cbs^–/–^* and *cbs^+/–^* mice compared to control *cbs^+/+^* mice (left panel), human retinal endothelial cells (HRECs) treated with homocysteine (Hcy) (middle panel) and retinal pigment epithelial cells ARPE-19 treated with Hcy (right panel) showed that Hcy induced a significant increase in DNMT activity (^*^p < 0.05, ^**^p < 0.01). (**B**) Measurement of Histone deacetylases (HDAC) enzyme activity in *cbs^–/–^* and *cbs^+/–^* mice compared to control *cbs^+/+^* mice (left panel), human retinal endothelial cells (HRECs) treated with homocysteine (Hcy) (middle panel) and retinal pigment epithelial cells ARPE-19 treated with Hcy (right panel) showed Hcy induced a significant increase in HDAC enzyme activity (^*^p < 0.05, ^**^p < 0.01, ^***^p < 0.001).

Experiments were performed (*in vivo* and *in vitro)* to evaluate the effect of HHcy on the activities of DNMT enzymes (Figure 1A) and HDAC enzymes (Figure 1B), as key enzymes of DNA methylation and histone deacetylation respectively. *In vitro* experiments using HRECs and ARPE-19 treated with Hcy showed a significant increase of DNMT and HDAC activities in a dose-dependent manner. This was consistent with the *in vivo* effect of HHcy, where both *cbs^+/–^* and *cbs^–/–^* mice retina showed marked increase of the activity of DNMT enzymes as well as HDAC enzymes compared to the normal control group.

### Inhibition of DNA methylation and histone deacetylation diminishes HHcy effect on mice retina

**Figure 2:**
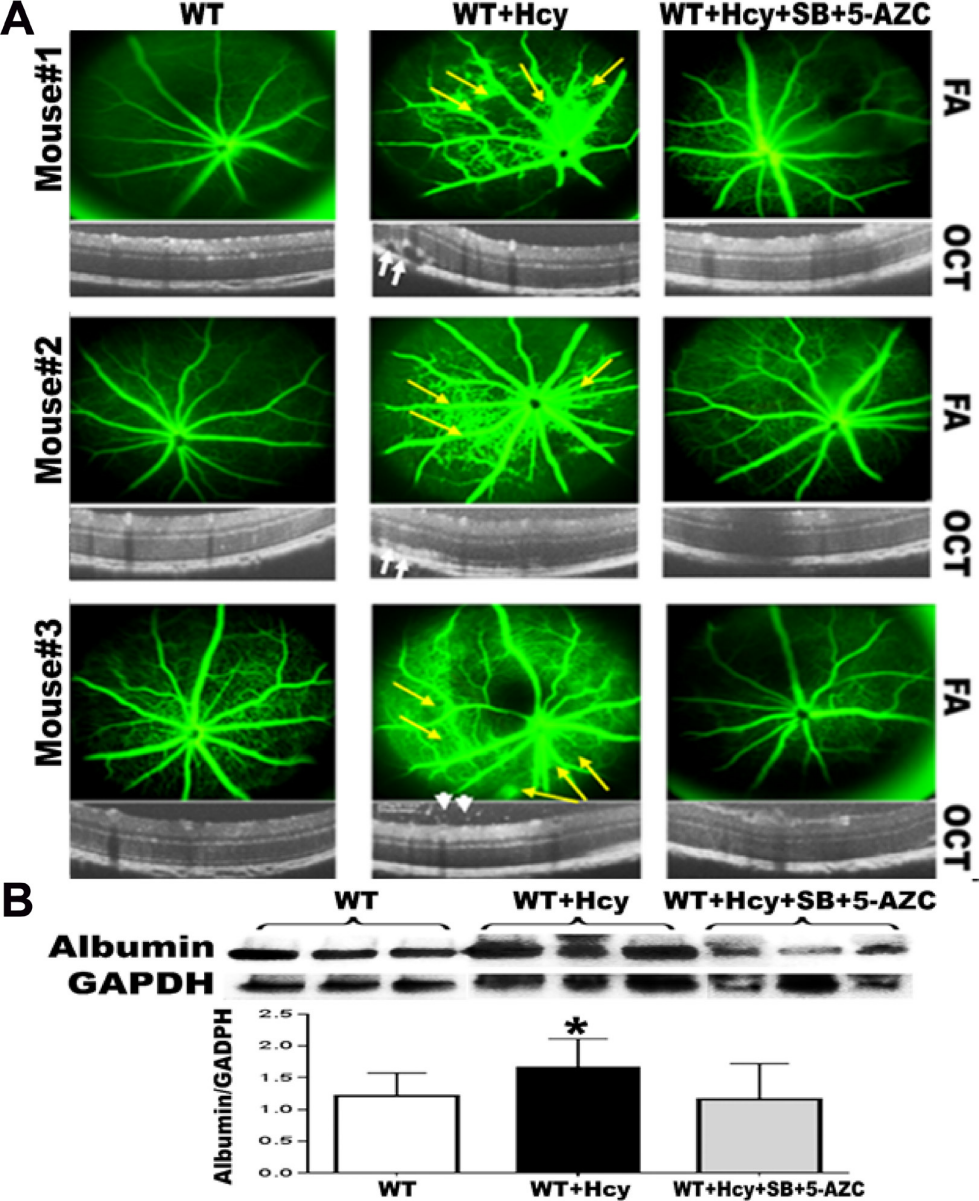
Effect of inhibition of DNA methylation and histone deacetylation on Hcy-induced BRB dysfunction OCT and FA examinations of 24-week-old mice injected intravitreally with Hcy with and without intraperitoneal injection of combined inhibitors of DNA methylation (DNMT inhibitor, 5-Azacytidine) and histone deacetylation (HDAC inhibitor, Sodium butyrate). The FA and OCT examination in the mice injected with Hcy revealed diffuse hyper fluorescence, leakage and focal areas of hyper-fluorescence coming from the deeper layer (yellow arrows) suggesting choroidal neovascularization (CNV). OCT examination revealed disruption of the retinal morphology with RPE changes with the development of CNV (white arrows) and intra-retinal neovascularization (arrow heads). Interestingly, combined injection of Hcy and HDAC&DNMT inhibitors protected the retina from the distributing effect of Hcy alone on mice retina as shown in both FA and OCT (**A**)The leakage was confirmed by measuring the albumin leakage in the retinas by western blotting (**B**), which was significantly increased in the Hcy-injected mice eyes compared to non-injected eyes and the eyes injected with Hcy along with intraperitoneal injection of combined DNMT&HDAC inhibitors, ^*^p < 0.05.

Optical coherence tomography (OCT) and fluorescein angiography (FA) examinations of 24 weeks living mice injected intravitreally with Hcy with and without intraperitoneal injection of combined inhibitors of DNA methylation (DNMT inhibitor, 5-Azacytidine) and histone deacetylation (HDAC inhibitor, Sodium butyrate) showed changes in the FA and OCT examination in the mice injected with Hcy similar to what has been reported before in our previous publication [2, 4]. FA revealed diffuse hyper fluorescence, leakage and focal areas of hyper-fluorescence coming from the deeper layer (yellow arrows) suggestive of choroidal neovascularization (CNV) and the OCT examination revealed disruption of the retinal morphology with RPE changes with the development of CNV (white arrows), and intra-retinal neovascularization (arrow heads). Interestingly, combined injection of Hcy and HDAC/DNMT inhibitors protected the retina from the distributing effect of Hcy alone on mice retina as shown in both FA and OCT (Figure 2)

### Differential expression profile of miRNAs in *cbs^–/–^ and cbs^+/–^* mice retina

**Figure 3:**
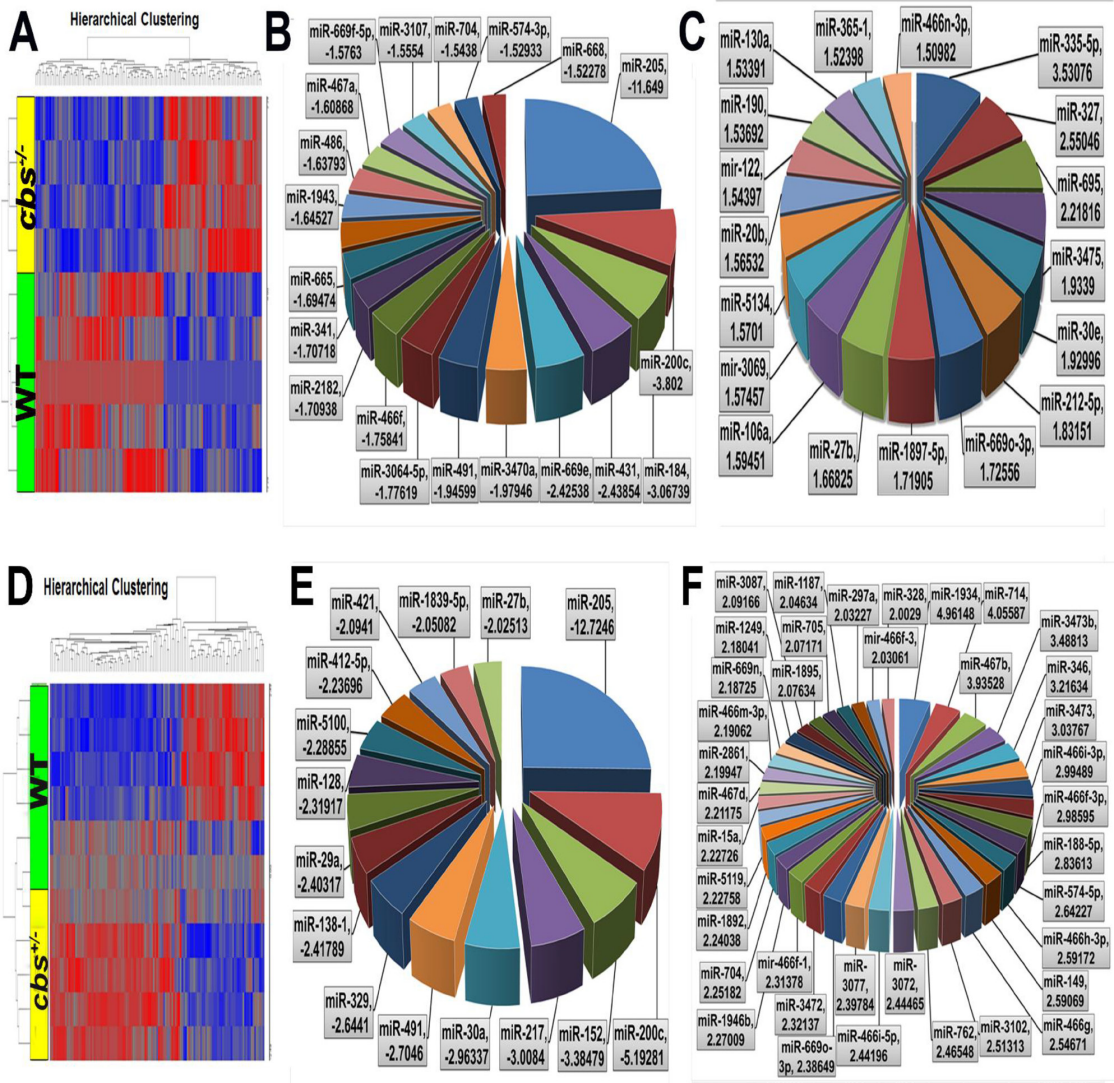
Differential expression profile of miRNAs in *cbs^–/–^ and cbs^+/–^* mouse retina (**A**) Heat map showing differentially expressed miRNAs in *cbs^–/–^* mouse retina compared to *cbs^+/+^* wild type control. Blue color indicates downregulated miRNAs and red color indicates upregulated miRNAs. (**B**) Pie chart showing miRNAs downregulated in *cbs^–/–^* mouse retina compared to *cbs^+/+^* wild type control (**C**) Pie chart showing miRNAs upregulated in *cbs^–/–^* mouse retina compared to *cbs^+/+^* wild type control. (**D**) Heat map showing differentially expressed miRNAs in *cbs^+/–^* mouse retina compared to *cbs^+/+^* wild type control. Blue color indicates downregulated miRNAs and red color indicates upregulated miRNAs. (**E**) Pie chart showing miRNAs downregulated in *cbs^+/–^* mouse retina compared to *cbs^+/+^* wild type control. (**F**) Pie chart showing miRNAs upregulated in *cbs^+/–^* mouse retina compared to *cbs^+/+^* wild type control.

In order to identify the impact of severely elevated Hcy levels on the retinal miRNA profile, a microarray analysis was done for retinas of *cbs^–/–^* mice compared to control *cbs^+/+^* retinas at 3 weeks old (Figure 3A–3C). The data was filtered by using *p* value less than 0.05 which reveals 217 miRNAs significantly differentially expressed versus *cbs^+/+^* (*p* value < 0.05) of which 124 were downregulated and 93 were upregulated. From these statistically significant miRNAs, 39 miRNAs were more than 1.5-fold change, 20 miRNAs of them were downregulated (Table 1A) and 19 were upregulated (Table 1B). In order to identify the impact of mild to moderate elevation of Hcy levels on the retinal miRNA profile, miRNA microarray analysis was done for the *cbs^+/–^* mice retina compared to the *cbs^+/+^* control (Figure 3D–3F). The differential miRNA expression profiling of the *cbs^+/–^* retinas showed 307 statistically significant and differentially expressed miRNAs compared to the *cbs^+/+^* control (157 miRNAs were downregulated and 150 were upregulated). Further filtration of these miRNAs based on the fold change cutoff more than 1.5, revealed that 127 miRNAs were significant with fold change more than 1.5; of which 49 were downregulated (Table 2A) and 78 were upregulated (Table 2B).

**Table 1A:** Downregulated miRNAs in cbs^–/–^

Species Scientific Name	Transcript ID	*p*-value (*cbs*^–/–^ vs. WT)	Fold-Change (*cbs^–/–^* vs. WT)
Musmusculus	mmu-miR-205_st	0.0031763	–11.649
Musmusculus	mmu-miR-200c_st	0.0246158	–3.802
Musmusculus	mmu-miR-184_st	0.0170031	–3.06739
Musmusculus	mmu-miR-431_st	0.00570439	–2.43854
Musmusculus	mmu-miR-669e_st	0.00155807	–2.42538
Musmusculus	mmu-miR-3470a_st	0.00672318	–1.97946
Musmusculus	mmu-miR-491_st	0.00328488	–1.94599
Musmusculus	mmu-miR-3064-5p_st	0.0276855	–1.77619
Musmusculus	mmu-miR-466f_st	0.00409442	–1.75841
Musmusculus	mmu-miR-2182_st	0.00532508	–1.70938
Musmusculus	mmu-miR-341_st	0.0497367	–1.70718
Musmusculus	mmu-miR-665-star_st	0.00544338	–1.69474
Musmusculus	mmu-miR-1943-star_st	0.0157678	–1.64527
Musmusculus	mmu-miR-486_st	0.0396356	–1.63793
Musmusculus	mmu-miR-467a-star_st	0.00240509	–1.60868
Musmusculus	mmu-miR-669f-5p_st	0.0107809	–1.5763
Musmusculus	mmu-miR-3107-star_st	0.0238135	–1.5554
Musmusculus	mmu-miR-704_st	0.0398611	–1.5438
Musmusculus	mmu-miR-574-3p_st	0.0192491	–1.52933
Musmusculus	mmu-miR-668_st	0.00500674	–1.52278

**Table 1B:** Upregulated miRNAs in cbs^−/−^

Species Scientific Name	Transcript ID	*p*-value (*cbs*^–/–^ vs. WT)	Fold-Change (*cbs^–/–^* vs. WT)
Musmusculus	mmu-miR-466n-3p_st	0.0190517	1.50982
Musmusculus	mmu-miR-365-1-star_st	0.0245969	1.52398
Musmusculus	mmu-miR-130a_st	0.00204146	1.53391
Musmusculus	mmu-miR-190-star_st	0.0134738	1.53692
Musmusculus	hp_mmu-mir-122_st	0.00397692	1.54397
Musmusculus	mmu-miR-20b_st	0.0346663	1.56532
Musmusculus	mmu-miR-5134_st	0.0469534	1.5701
Musmusculus	hp_mmu-mir-3069_st	0.0492988	1.57457
Musmusculus	mmu-miR-106a_st	0.0113837	1.59451
Musmusculus	mmu-miR-27b-star_st	0.0196148	1.66825
Musmusculus	mmu-miR-1897-5p_st	0.0257959	1.71905
Musmusculus	mmu-miR-669o-3p_st	0.00317962	1.72556
Musmusculus	mmu-miR-212-5p_st	0.001819	1.83151
Musmusculus	mmu-miR-30e_st	0.00214344	1.92996
Musmusculus	mmu-miR-3475_st	0.0178601	1.9339
Musmusculus	mmu-miR-695_st	0.0285587	2.21816
Musmusculus	mmu-miR-327_st	0.0307551	2.55046
Musmusculus	mmu-miR-335-5p_st	0.00904379	3.53076

**Table 2A:** Downregulated miRNAs in cbs^+/−^

Species Scientific Name	Transcript ID	*p*-value (*cbs*^+/–^ vs. WT)	Fold-Change (*cbs*^+/–^ vs. WT)
Musmusculus	mmu-miR-205_st	0.00222063	–12.7246
Musmusculus	mmu-miR-200c_st	0.00648219	–5.19281
Musmusculus	mmu-miR-152_st	0.00458781	–3.38479
Musmusculus	mmu-miR-217_st	0.000331015	–3.0084
Musmusculus	mmu-miR-30a-star_st	0.0228718	–2.96337
Musmusculus	mmu-miR-491_st	0.000552044	–2.7046
Musmusculus	mmu-miR-329_st	0.0117443	–2.6441
Musmusculus	mmu-miR-138-1-star_st	0.00500405	–2.41789
Musmusculus	mmu-miR-29a_st	0.03658	–2.40317
Musmusculus	mmu-miR-128_st	0.00381972	–2.31917
Musmusculus	mmu-miR-5100_st	7.60E-05	–2.28855
Musmusculus	mmu-miR-412-5p_st	0.0241069	–2.23696
Musmusculus	mmu-miR-421_st	0.00638554	–2.0941
Musmusculus	mmu-miR-1839-5p_st	0.00541099	–2.05082
Musmusculus	mmu-miR-27b_st	0.00384257	–2.02513
Musmusculus	mmu-miR-674-star_st	0.0249087	–1.97604
Musmusculus	mmu-miR-362-5p_st	0.00180754	–1.87892
Musmusculus	mmu-miR-543_st	0.0356474	–1.84637
Musmusculus	mmu-miR-154_st	0.0155685	–1.8344
Musmusculus	mmu-miR-132_st	0.00450186	–1.81842
Musmusculus	mmu-miR-431_st	0.0149049	–1.79581
Musmusculus	mmu-miR-338-5p_st	0.0192303	–1.79459
Musmusculus	mmu-miR-212-3p_st	0.0189292	–1.79255
Musmusculus	mmu-miR-194_st	0.0331242	–1.78064
Musmusculus	mmu-miR-100_st	0.00951479	–1.78007
Musmusculus	mmu-miR-129-2-3p_st	0.00833425	–1.75675
Musmusculus	mmu-miR-412-3p_st	0.0322016	–1.75503
Musmusculus	mmu-miR-99a_st	0.000217283	–1.75086
Musmusculus	mmu-miR-126-3p_st	0.00834968	–1.74797
Musmusculus	mmu-miR-28_st	0.0256688	–1.74017
Musmusculus	mmu-miR-138-2-star_st	0.0412741	–1.72159
Musmusculus	mmu-miR-434-3p_st	0.00699578	–1.7189
Musmusculus	mmu-miR-29b-2-star_st	0.0314635	–1.71233
Musmusculus	mmu-miR-379_st	0.00225407	–1.69645
Musmusculus	mmu-miR-673-3p_st	0.0141924	–1.67801
Musmusculus	mmu-miR-26b_st	0.0347711	–1.67488
Musmusculus	mmu-miR-375_st	0.0115361	–1.65935
Musmusculus	mmu-miR-150_st	0.014412	–1.65243
Musmusculus	mmu-miR-3064-5p_st	0.0258966	–1.6452
Musmusculus	mmu-miR-495_st	0.0197081	–1.62409
Musmusculus	mmu-miR-485-star_st	0.015586	–1.60495
Musmusculus	mmu-miR-874_st	0.0419194	–1.59787
Musmusculus	mmu-miR-487b_st	0.0181579	–1.59098
Musmusculus	mmu-miR-212-5p_st	0.00878037	–1.58842
Musmusculus	mmu-miR-30c-2-star_st	0.0107089	–1.54934
Musmusculus	mmu-miR-22_st	0.000198259	–1.54389
Musmusculus	mmu-miR-30c_st	0.0029497	–1.54025
Musmusculus	mmu-miR-195_st	0.00855184	–1.53897
Musmusculus	mmu-miR-342-3p_st	0.000129999	–1.52217

**Table 2B:** Upregulated miRNAs in cbs+/−

Species Scientific Name	Transcript ID	*p*-value (*cbs*^+/–^ vs. WT)	Fold-Change (*cbs*^+/–^ vs. WT)
Musmusculus	hp_mmu-mir-297a-6_x_st	0.00478449	1.51015
Musmusculus	hp_mmu-mir-692-1_x_st	0.029077	1.57678
Musmusculus	mmu-miR-466a-3p_st	0.0331968	1.59417
Musmusculus	mmu-miR-5110_st	0.0112957	1.5946
Musmusculus	mmu-miR-5107_st	0.000824498	1.59638
Musmusculus	mmu-miR-696_st	0.0391273	1.5983
Musmusculus	mmu-miR-5109_st	0.00162405	1.60236
Musmusculus	mmu-miR-467h_st	0.0229014	1.62104
Musmusculus	hp_mmu-mir-466m_x_st	0.0375686	1.63873
Musmusculus	mmu-miR-92b-star_st	0.00583904	1.64273
Musmusculus	mmu-miR-669c_st	0.00218886	1.64946
Musmusculus	hp_mmu-mir-194-2_st	0.00178807	1.65489
Musmusculus	mmu-miR-466h-5p_st	0.0237521	1.67801
Musmusculus	mmu-miR-5126_st	0.000430848	1.68182
Musmusculus	mmu-miR-211-star_st	0.00584196	1.6863
Musmusculus	mmu-miR-5128_st	0.000296384	1.70787
Musmusculus	mmu-miR-669b_st	0.0136101	1.71598
Musmusculus	mmu-miR-344e-star_st	0.0154884	1.72918
Musmusculus	mmu-miR-3470b_st	0.0130989	1.73068
Musmusculus	mmu-miR-504-star_st	0.0058915	1.74408
Musmusculus	mmu-miR-3082-5p_st	0.0111523	1.76029
Musmusculus	hp_mmu-mir-466f-2_x_st	0.014706	1.76379
Musmusculus	hp_mmu-mir-1194_st	0.00469116	1.7654
Musmusculus	mmu-miR-1946a_st	0.00573744	1.77638
Musmusculus	mmu-miR-669m-5p_st	0.00277103	1.77932
Musmusculus	mmu-miR-5130_st	0.00104483	1.78517
Musmusculus	mmu-miR-1896_st	0.00488995	1.80244
Musmusculus	mmu-miR-669k-star_st	0.016768	1.80843
Musmusculus	mmu-miR-466f-5p_st	0.0019437	1.82175
Musmusculus	mmu-miR-690_st	0.0118474	1.83076
Musmusculus	mmu-miR-3960_st	0.00349971	1.83654
Musmusculus	mmu-miR-466f_st	0.00468465	1.83736
Musmusculus	mmu-miR-669h-3p_st	0.0478939	1.85694
Musmusculus	mmu-miR-467f_st	0.0161899	1.85717
Musmusculus	mmu-miR-1894-3p_st	0.000747658	1.87084
Musmusculus	mmu-miR-3104-5p_st	0.000782604	1.9097
Musmusculus	mmu-miR-3102-5p.2_st	0.00260383	1.93142
Musmusculus	mmu-miR-466m-5p_st	0.0090442	1.93389
Musmusculus	mmu-miR-698_st	0.0341733	1.95773
Musmusculus	mmu-miR-466q_st	0.0332336	1.98251
Musmusculus	mmu-miR-328-star_st	0.000301474	2.0029
Musmusculus	hp_mmu-mir-466f-3_x_st	0.0197357	2.03061
Musmusculus	mmu-miR-297a_st	0.0413891	2.03227
Musmusculus	mmu-miR-1187_st	0.00682685	2.04634
Musmusculus	mmu-miR-705_st	0.000880031	2.07171
Musmusculus	mmu-miR-1895_st	0.00234172	2.07634
Musmusculus	mmu-miR-3087-star_st	0.00362349	2.09166
Musmusculus	mmu-miR-1249-star_st	0.0455887	2.18041
Musmusculus	mmu-miR-669n_st	0.00800571	2.18725
Musmusculus	mmu-miR-466m-3p_st	0.0484292	2.19062
Musmusculus	mmu-miR-2861_st	0.00458595	2.19947
Musmusculus	mmu-miR-467d-star_st	0.0340084	2.21175
Musmusculus	mmu-miR-15a-star_st	0.015554	2.22726
Musmusculus	mmu-miR-5119_st	0.00943229	2.22758
Musmusculus	mmu-miR-1892_st	0.00173456	2.24038
Musmusculus	mmu-miR-704_st	0.0138606	2.25182
Musmusculus	mmu-miR-1946b_st	0.00187268	2.27009
Musmusculus	hp_mmu-mir-466f-1_x_st	0.00485862	2.31378
Musmusculus	mmu-miR-3472_st	0.00581812	2.32137
Musmusculus	mmu-miR-669o-3p_st	0.0372755	2.38649
Musmusculus	mmu-miR-3077-star_st	0.00085742	2.39784
Musmusculus	mmu-miR-466i-5p_st	0.00281848	2.44196
Musmusculus	mmu-miR-3072-star_st	0.000883966	2.44465
Musmusculus	mmu-miR-762_st	0.000828458	2.46548
Musmusculus	mmu-miR-3102-star_st	1.78E-05	2.51313
Musmusculus	mmu-miR-466g_st	0.000631176	2.54671
Musmusculus	mmu-miR-149-star_st	0.000486972	2.59069
Musmusculus	mmu-miR-466h-3p_st	0.0010598	2.59172
Musmusculus	mmu-miR-574-5p_st	0.00398314	2.64227
Musmusculus	mmu-miR-188-5p_st	0.00878782	2.83613
Musmusculus	mmu-miR-466f-3p_st	0.00355105	2.98595
Musmusculus	mmu-miR-466i-3p_st	0.0141958	2.99489
Musmusculus	mmu-miR-3473_st	0.00510505	3.03767
Musmusculus	mmu-miR-346-star_st	0.000254779	3.21634
Musmusculus	mmu-miR-3473b_st	0.00306257	3.48813
Musmusculus	mmu-miR-467b-star_st	0.0059904	3.93528
Musmusculus	mmu-miR-714_st	0.00335002	4.05587
Musmusculus	mmu-miR-1934-star_st	0.000496819	4.96148

### Comparison of the common differentially expressed miRNAs in *cbs^–/–^* & *cbs^+/–^* and WT& diabetic (STZ) mouse retina

**Figure 4:**
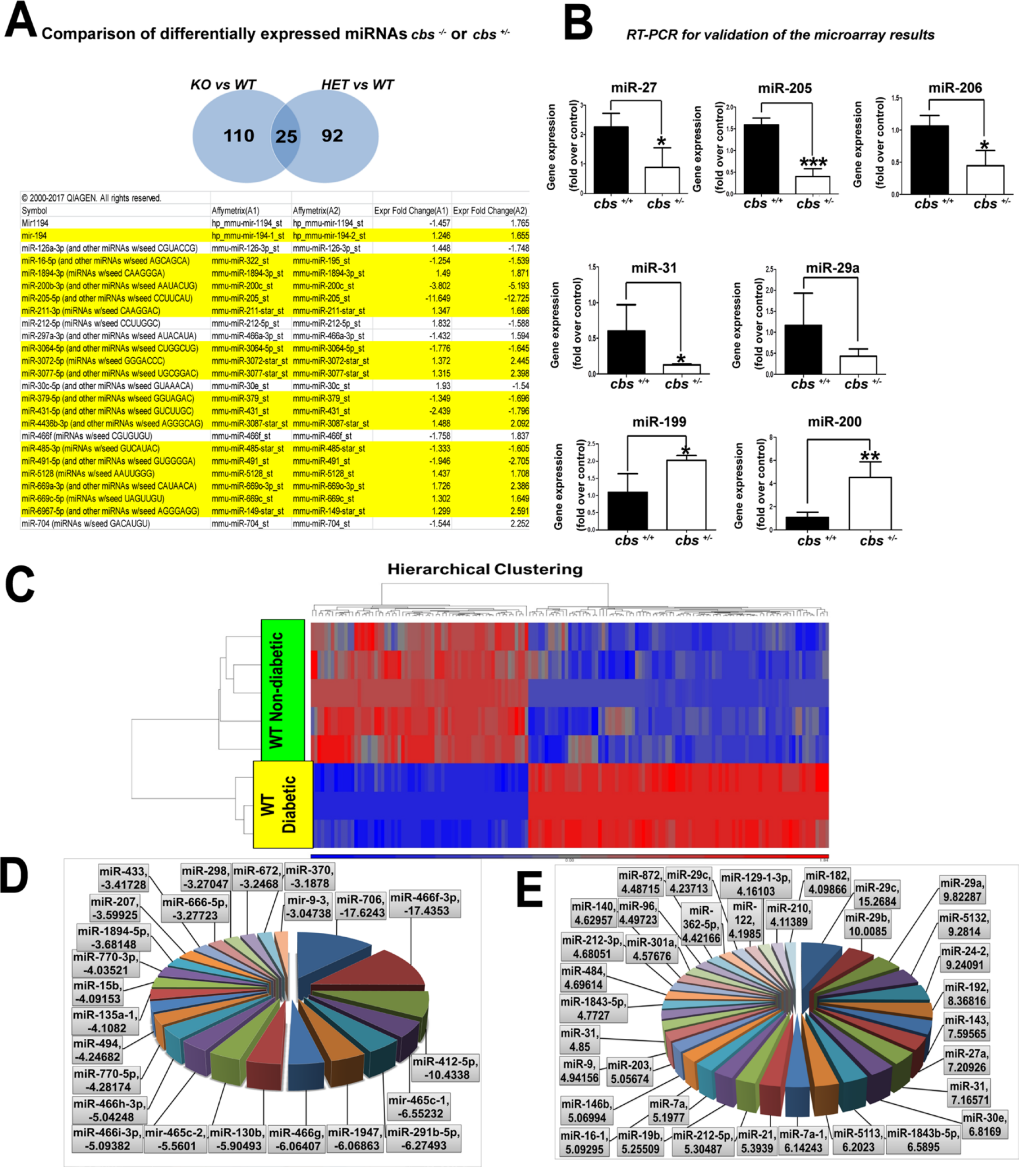
(**A**) Common miRNAs differentially expressed in *cbs^–/–^* or *cbs^+/–^*. IPA shows 25 miRNAs were commonly changed in both *cbs^–/–^* or *cbs^+/–^*. 18 miRNA (highlighted in yellow) were differentially expressed in a consistent manner between the 2 groups. (**B**) RT-PCR for validation of microarray results of miRNAs differentially expressed in *cbs^–/–^* or *cbs^+/–^* miRNAs (miR-205, miR-206 and miR-27, miR-31) were significantly downregulated in *cbs^+/–^* in comparison with the control. MiR-29a showed non-significant downregulation (^*^p < 0.05, ^***^p < 0.001). MiRNAs (miR-199 and miR-200) were significantly upregulated in *cbs^+/–^* in comparison with the control (^*^p < 0.05, ^**^p < 0.01).Diabetes induced alterations of miRNA differential profile in mouse retina. (**C**) Heat map showing differentially expressed miRNAs in diabetic (STZ) mouse retina comparison with WT non-diabetic retina. Blue color indicates downregulated miRNAs and red color indicates upregulated miRNAs. (**D**) Pie chart showing miRNAs downregulated in diabetic mouse retina. (**E**) Pie chart showing miRNAs upregulated in diabetic mouse retina.

In order to explore miRNAs that are commonly changed with either complete or partial deletion of the *cbs* enzyme, data was loaded into the IPA (Ingenuity Pathway Analysis) for comparison analysis. The comparison of the 2 data sets showed the presence of 25 miRNAs that were commonly changed in both *cbs^–/–^* or *cbs^+/–^* mouse retina. Out of these 25 miRNAs, 18 miRNAs were differentially expressed in a consistent manner between the 2 groups (Figure 4A, highlighted); 8 miRNAs were downregulated in both groups (miR-16, miR-200, miR-205, miR-3064, miR-379, miR-431, miR-485 and miR-491) and 10 miRNAs were upregulated in both groups (miR-194, miR-1894, miR-211, miR-3072, miR- 3077, miR-4436, miR-5128, miR-669a, miR-669c and miR-6967). Microarray data results were validated by subjecting retinal RNA samples from *cbs^+/–^* and *cbs^+/+^* mice to RT-PCR analysis (Figure 4B). Consistently with the microarray results, miR-205 (*p* value = 0.001), miR-206 (*p* value = 0.01) and miR-27 (*p* value = 0.04) were significantly downregulated in *cbs^+/–^* compared to control *cbs^+/+^* (*p* value < 0.05). MiR-29a showed a tendency of downregulation but it did not reach the statistical significance. However, miR-199 (*p* value = 0.01) and miR-200 (*p* value = 0.004) in contrary to the microarray results were significantly upregulated in *cbs^+/–^* in comparison with the control *cbs^+/+^* (*p* value < 0.05). In contrast, miR-31 was upregulated in the microarray data but was downregulated with RT-PCR validation (*p* value = 0.04). U6 was used as an internal control.

### Differential expression profile of retinal miRNAs during diabetes

We aimed to detect the differential expression of retinal miRNAs under the influence of diabetes (Figure 4C–4E). WT animals were treated with streptozotocin (STZ) 50 mg/kg for five consecutive days. Diabetes was confirmed by measuring blood glucose level (mice with blood glucose level > 250 mg/dl were considered diabetic). Microarray analysis was performed for diabetic retinal RNA in comparison with the non-diabetic controls. 312 miRNAs were found to be changed after 1.5-fold change cutoff and *p* value < 0.05 (129 miRNAs were downregulated and 183 miRNAs were upregulated).

### Shared miRNAs among *cbs^–/–^*, *cbs^+/–^* and diabetic mouse retina

**Figure 5:**
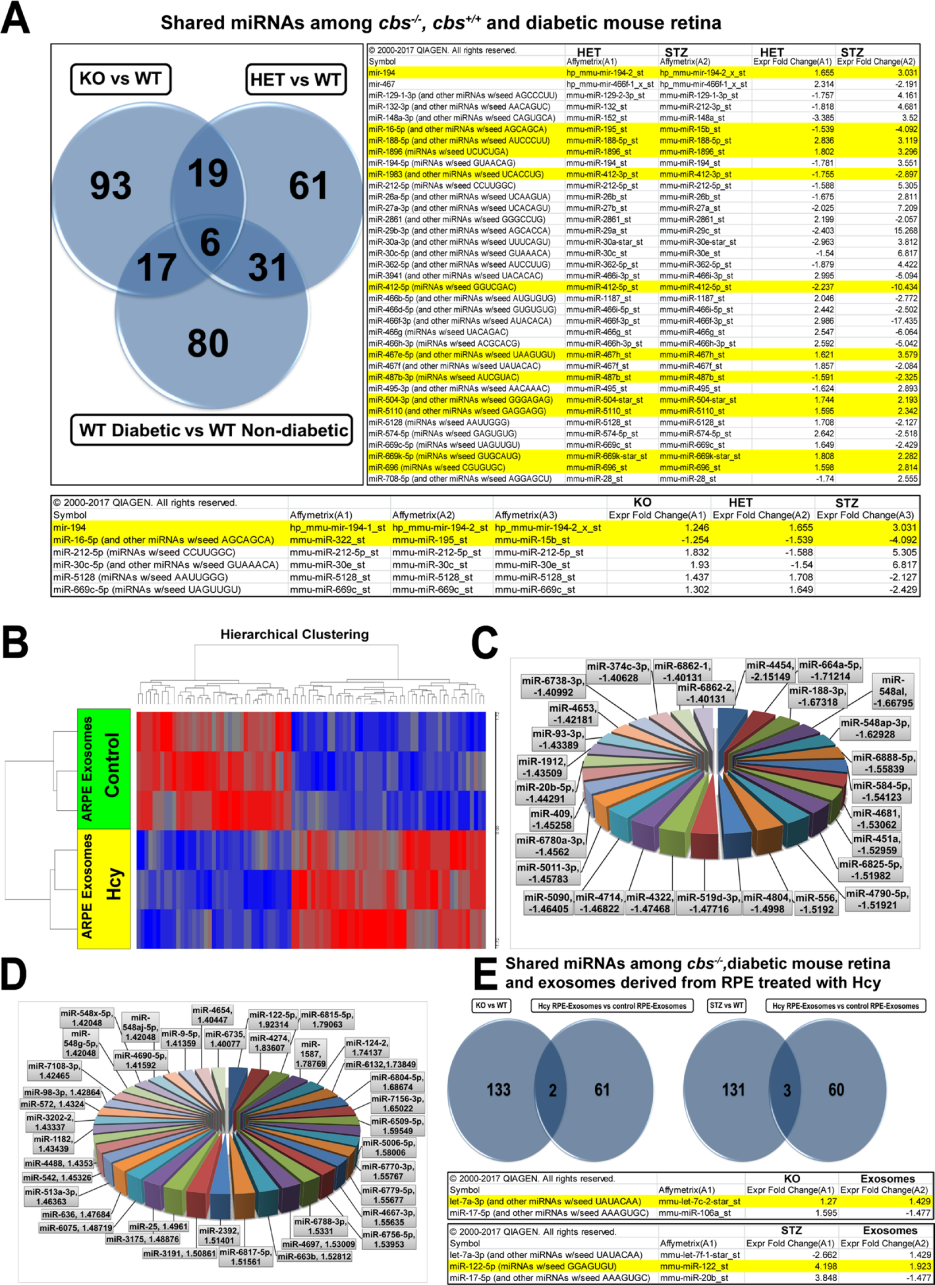
(**A**) Common miRNAs differentially expressed in *cbs^–/–^* or *cbs^+/–^* and diabetic mouse retina. Comparison showed 37 miRNAs commonly changed in both *cbs^+/–^* and diabetic retinas. 12 miRNA (highlighted in yellow) were differentially expressed in a consistent manner between *cbs^+/–^* and diabetic groups. PA triple comparison showed 6 miRNAs that were commonly changed among *cbs^–/–^* , *cbs^+/–^* or diabetic mouse retina. Two miRNAs were differentially expressed in a consistent manner between the 3 groups. Differential expression profile of miRNAs in exosomes isolated from ARPE cells treated with Hcy. (**B**) Heat map showing differentially expressed miRNAs in exosomes isolated from ARPE cells treated with Hcy (100 μM for 24 hours) in comparison with exosomes isolated from vehicle treated cells. Blue color indicates downregulated miRNAs and red color indicates upregulated miRNAs. (**C**) Pie chart showing miRNAs downregulated in exosomes isolated from ARPE cells treated with Hcy. (**D**) Pie chart showing miRNAs upregulated in exosomes isolated from ARPE cells treated with Hcy. (**E**) Common miRNAs differentially expressed in *cbs^–/–^,* diabetic mouse retina and exosomes released from ARPE treated with Hcy. The left Venn diagram shows that 2 miRNAs were commonly differentially expressed in ARPE-exosomes and *cbs^–/–^* (miR-17-5p and let-7a-3p). The right Venn diagram shows that 3miRNAs were commonly differentially expressed in ARPE-exosomes and diabetic mouse retina (miR-17-5p, let-7a-3p and miR-122-5p).

To investigate the similarity in alteration of retinal miRNAs profile between HHcy and diabetes, IPA software was used to compare differential retinal miRNA profiles in *cbs^+/–^* vs the diabetic retinas (Figure 5A). The analysis showed that 37 miRNAs were commonly changed in both conditions. In the microarray data, 12 miRNAs were consistent in their change either with HHcy or diabetes; of which 4 miRNAs were downregulated in both groups (miR-16, miR-1983, miR-412 and miR-487) and 8 miRNAs (miR-194, miR-188, miR-1896, miR-467e, miR-504, miR-5110, miR-669k and miR-696) were upregulated in both groups. A triple comparison was also done that included *cbs^–/–^*, *cbs^+/–^* and STZ retinas, which revealed 6 miRNAs (miR-194, miR-16, miR-212, miR-30c, miR-5128 and miR-669c) that were commonly changed among *cbs^–/–^*, *cbs^+/–^* and diabetes; 2 of these miRNAs were consistently changed among the three groups (miR-194 was upregulated and miR-16 was downregulated).

### HHcy induces alteration of miRNAs similar to AMD

We have reported that HHcy induces AMD-like changes in mouse retina [4]. Therefore, the current work aimed to check if HHcy induces changes in miRNAs similar to what have been reported to be implicated in the development of AMD [69]. Interestingly, miRNAs have also been shown to play critical roles in different pathways in a laser-induced choroidal neovascularization mouse model [70]. miR-205, miR-27, miR-29 and miR-31 were significantly changed in our *cbs^+/–^* retina microarray and were also reported to be involved in AMD. The study revealed downregulation of miR-205, miR-27, miR-31, and miR-29 in the cbs*^+/–^* retinas. MiR-31 was reported to be significantly decreased in an ischemia-induced mouse model of retinal neovascularization and in a laser-induced mouse model of choroidal neovascularization in the absence of ischemia [71]. While intraocular injection with pre-miR-31 significantly reduced the size of the choroidal neovascular lesions. A recent study also demonstrated that knockdown of miR-27, which downregulates the antiangiogenic factors Sprouty2 and semaphorin 6A (Sema6A), is protective against laser-induced choroidal neovascularization [70].

### Differentially expressed miRNAs in exosomes isolated from Hcy-treated ARPE

Exosomes released from RPE represent a very important and innovative way of retinal intercellular communications. The outer blood-retinal barrier integrity is formed by tight junctions among retinal pigment epithelial cells [72, 73]. The outer BRB is mainly affected in AMD. To investigate if HHcy induces changes in the miRNA cargo of RPE-released exosomes as a possible early epigenetic modification tool that can impact the retinal barrier function, conditioned media of ARPE-19 cells treated with Hcy 100 μM for 24 hr was collected and exosomes were isolated using the appropriate protocol. The concentration and size of the isolated exosomes were determined using NTA as indicated in the methods section. Total extracted RNA from the isolated exosomes and purity of the extracted RNA was validated using the Bioanalyzer chip (Supplementary Figure 1). Following exosomal RNA isolation, miRNA microarray analysis was performed. The analysis revealed that Hcy altered RPE-exosomal-miRNA content. 88 miRNAs were identified (39 miRNAs were downregulated and 49 miRNAs were upregulated) with1.4-fold change cutoff and *p* < 0.05 (Figure 5B–5D and Table 3A and 3B)

**Table 3A:** Downregulated miRNAs inARPE- derived exosomes

Species Scientific Name	Transcript ID	*p*-value (HCY vs. ARPE_cont)	Fold-Change (HCY vs. ARPE_cont)
Homo sapiens	hsa-miR-4454	0.00806874	–2.15149
Homo sapiens	hsa-miR-664a-5p	0.00933842	–1.71214
Homo sapiens	HBII-316	0.0371439	–1.6763
Homo sapiens	hsa-miR-188-3p	0.0050997	–1.67318
Homo sapiens	hsa-mir-548al	0.0409855	–1.66795
Homo sapiens	hsa-miR-548ap-3p	0.00270826	–1.62928
Homo sapiens	hsa-miR-6888-5p	0.0244117	–1.55839
Homo sapiens	hsa-miR-584-5p	0.00101538	–1.54123
Homo sapiens	hsa-miR-4681	0.0273394	–1.53062
Homo sapiens	hsa-miR-451a	0.0147813	–1.52959
Homo sapiens	HBI-36	0.0477323	–1.51987
Homo sapiens	hsa-miR-6825-5p	0.0137077	–1.51982
Homo sapiens	hsa-miR-4790-5p	0.0229827	–1.51921
Homo sapiens	hsa-mir-556	0.00579586	–1.5192
Homo sapiens	hsa-mir-4804	0.018718	–1.4998
Homo sapiens	U73a	0.0395006	–1.4917
Homo sapiens	hsa-miR-519d-3p	0.00303684	–1.47716
Homo sapiens	hsa-mir-4322	0.0277639	–1.47468
Homo sapiens	hsa-mir-4714	0.02808	–1.46822
Homo sapiens	U94	0.0279962	–1.46617
Homo sapiens	hsa-mir-5090	0.0181336	–1.46405
Homo sapiens	hsa-miR-5011-3p	0.010633	–1.45783
Homo sapiens	hsa-miR-6780a-3p	0.04771	–1.4562
Homo sapiens	hsa-mir-409	0.0371396	–1.45258
Homo sapiens	hsa-miR-20b-5p	0.0469542	–1.44291
Homo sapiens	hsa-miR-1912	0.00969643	–1.43509
Homo sapiens	hsa-miR-93-3p	0.023408	–1.43389
Homo sapiens	hsa-mir-4653	0.0162663	–1.42181
Homo sapiens	hsa-miR-6738-3p	0.0410678	–1.40992
Homo sapiens	hsa-miR-374c-3p	0.0481363	–1.40628
Homo sapiens	hsa-mir-6862-1	0.0233446	–1.40131
Homo sapiens	hsa-mir-6862-2	0.0233446	–1.40131

**Table 3B:** Upregulated miRNAs in ARPE- derived exosomes

Species Scientific Name	Transcript ID	*p*-value (HCY vs. ARPE_cont)	Fold-Change (HCY vs. ARPE_cont)
Homo sapiens	hsa-mir-6735	0.0419018	1.40077
Homo sapiens	hsa-mir-4654	0.0469582	1.40447
Homo sapiens	hsa-miR-9-5p	0.0216397	1.41359
Homo sapiens	hsa-miR-4690-5p	0.0403177	1.41592
Homo sapiens	hsa-miR-548g-5p	0.0342995	1.42048
Homo sapiens	hsa-miR-548x-5p	0.0342995	1.42048
Homo sapiens	hsa-miR-548aj-5p	0.0342995	1.42048
Homo sapiens	hsa-miR-7108-3p	0.0429707	1.42465
Homo sapiens	hsa-miR-98-3p	0.021263	1.42864
Homo sapiens	hsa-mir-572	0.0333677	1.4324
Homo sapiens	hsa-mir-3202-2	0.00783197	1.43337
Homo sapiens	hsa-mir-1182	0.0404323	1.43439
Homo sapiens	hsa-mir-4488	0.0230517	1.4353
Homo sapiens	hsa-mir-542	0.0407305	1.45326
Homo sapiens	hsa-miR-513a-3p	0.0100636	1.46363
Homo sapiens	hsa-miR-636	0.0252418	1.47684
Homo sapiens	hsa-miR-6075	0.016306	1.48719
Homo sapiens	hsa-mir-3175	0.0286791	1.48876
Homo sapiens	hsa-mir-25	0.0285173	1.4961
Homo sapiens	hsa-mir-3191	0.0252016	1.50861
Homo sapiens	hsa-miR-2392	0.0117701	1.51401
Homo sapiens	hsa-miR-6817-5p	0.0422369	1.51561
Homo sapiens	hsa-mir-663b	0.0437883	1.52812
Homo sapiens	hsa-mir-4697	0.0335516	1.53009
Homo sapiens	hsa-miR-6788-3p	0.0261082	1.5331
Homo sapiens	hsa-miR-6756-5p	0.00965285	1.53953
Homo sapiens	hsa-miR-4667-3p	0.0251413	1.55635
Homo sapiens	hsa-miR-6779-5p	0.0277697	1.55677
Homo sapiens	hsa-miR-6770-3p	0.0268935	1.55767
Homo sapiens	hsa-miR-5006-5p	0.0408652	1.58006
Homo sapiens	hsa-miR-6509-5p	0.0408825	1.59549
Homo sapiens	hsa-miR-7156-3p	0.0350715	1.65022
Homo sapiens	hsa-miR-6804-5p	0.00930951	1.68674
Homo sapiens	hsa-miR-6132	0.0261594	1.73849
Homo sapiens	hsa-mir-124-2	0.029337	1.74137
Homo sapiens	hsa-miR-1587	0.00634675	1.78769
Homo sapiens	hsa-miR-6815-5p	0.0313742	1.79063
Homo sapiens	hsa-miR-4274	0.0421541	1.83607
Homo sapiens	hsa-miR-122-5p	0.0478945	1.92314

### Comparison for shared miRNAs among *cbs^–/–^*, diabetic mouse retina and exosomes released from ARPE treated with Hcy

Different datasets were compared to check if there was a correlation between exosomal-miRNAs released from ARPE under the influence of Hcy and miRNAs of either *cbs^–/–^* or diabetic mouse retina. The comparison showed that ARPE-exosomes shared 2 differentially expressed miRNAs with *cbs^–/–^* (miR-17-5p and let-7a-3p) and shared 3 differentially expressed miRNAs with diabetic retina (miR-17-5p, let-7a-3p and miR-122-5p) (Figure 5E).

### IPA (ingenuity pathway analysis)

IPA showed that differentially expressed *cbs^+/–^* retinal miRNAs are involved in metabolic pathways, suggesting new epigenetically affected mechanisms (as ER stress, oxidative stress, autophagy and tight junctions signaling pathways). The analysis showed miRNAs that were related to ER stress pathway (let-7f, miR-351, miR-127, miR-133a, miR-195, miR-214 and miR-503), suggesting *CASP3*, *CASP7*, *XBP1*, *ATF6* and *ATF4* as possible target genes for these miRNAs (Table 4). Furthermore, the pathway analysis links a group of miRNAs that were differentially expressed in *cbs^+/–^* retina to oxidative stress pathway such as miR-205, miR-206, miR-217, miR-30, miR-27, miR-214 and miR-3473. These miRNAs target genes that are involved in the oxidative stress pathway such as *PRKCE*, *IRS1*, *HACD3*, *DNAJB1*, *FGFR3*, *PIK3R2*, *AKT1* and *PTPN11* as we can see in (Table 4). Other miRNAs were linked to the hypoxia signaling pathway, for instance, miR-205, miR-214, miR-217, miR-27, miR-29, miR-30 and miR-31. These miRNAs are targeting genes implicated in hypoxia signaling such as *PTEN*, *VEGFA*, *ATF4*, *IRS1*, *PIK3R1* and *HIF1A* (Table 5). Autophagy is emerging as a critical player in the pathogenesis of diseases like DR and AMD [74, 75]. Our data analysis revealed the association of some of the differentially expressed miRNAs in the *cbs^+/–^* retina with the autophagy pathway. MiR-206, miR-133, miR-199, miR-100 and miR-195 were implicated in the autophagy pathway targeting *BCL2*, *MTOR* and *SQSTM1* as possible autophagy gene targets (Table 6). Hcy also induces alteration of miRNAs related to tight junctions signaling such as miR-128, miR-132, miR-133, miR-195, miR-3473, miR-19, miR-200, miR-205, miR-214, miR-217, miR-23, miR-26, miR-29, miR-30, miR-31 AND miR-690. These miRNAs can target *TJP1*, *TGFBR1*, *RHOA*, *CLDN12*, *PTEN* and *CDC42* which are involved in the tight junctions signaling pathway (Table 7). Moreover, the IPA analysis showed miRNAs that differentially expressed in *cbs^+/–^* retina being involved in VEGF signaling, apoptosis, and gap junctions signaling (Table 8).

**Table 4:** Pathway analysis 1

miRNA (HET)	Target genes	Fold change
**miRNAs related to ER stress**		
mmu-let-7f_st	CASP3	–1.552
mmu-miR-351_st	CASP7	–1.499
mmu-miR-127_st	XBP1	–1.41
mmu-miR-133a_st	CASP9	–3.717
mmu-miR-195_st	ATF6	–1.539
mmu-miR-195_st	HSP90B1	–1.539
mmu-miR-214_st	ATF4	–2.004
mmu-miR-503_st	ATF6	1.256
**miRNAs related to Nrf2 related oxidative stress**		
mmu-miR-205_st	PRKCE	–12.725
mmu-miR-206_st	DNAJB1, HACD3, PPIB	–4.864
mmu-miR-217_st	IRS1	–3.008
mmu-miR-30a-star_st	PIK3C2A	–2.963
mmu-miR-29a_st	PIK3R1	–2.403
mmu-miR-27b_st	GRB2	–2.025
mmu-miR-214_st	ATF4	–2.004
mmu-miR-100_st	FGFR3	–1.78
mmu-miR-126-3p_st	IRS1, PIK3R2	–1.748
mmu-miR-150_st	BACH1	–1.652
mmu-miR-487b_st	MAP2K4	–1.591
mmu-let-7f_st	HMOX1, HRAS, KRAS, NRAS, Ras	–1.552
mmu-miR-30c_st	JUN	–1.54
mmu-miR-195_st	DNAJB4, FGFR1, GRB2, GSTM4, HMOX1, JUN, JUN/JUNB/JUND, MAP2K1, MAP2K4, MAPK3, RAF1, SQSTM1	–1.539
mmu-miR-351_st	MAP2K7	–1.499
mmu-miR-449a_st	MAP2K1	–1.416
mmu-miR-25_st	MAP2K4	–1.394
mmu-miR-146a_st	TLR9	–1.33
mmu-miR-494_st	HMOX1	1.377
mmu-miR-335-5p_st	PTPN11	1.997
mmu-miR-3473_st	AKT1	3.038

**Table 5:** Pathway analysis 2

miRNA (HET)	Target genes	Fold change
**miRNAs related to HIF1a and Hypoxia**		
mmu-let-7f_st	CSNK1D, HRAS, KRAS, NRAS, Ras	–1.552
mmu-miR-100_st	FGFR3	–1.78
mmu-miR-125b-2-3p_st	TP53	–1.495
mmu-miR-351_st	TP53, UBE2I	–1.499
mmu-miR-126-3p_st	IRS1, PIK3R2, VEGFA	–1.748
mmu-miR-132_st	MMP9	–1.818
mmu-miR-146a_st	MMP16, NOS2, TLR9	–1.33
mmu-miR-150_st	VEGFA	–1.652
mmu-miR-195_st	FGFR1, GRB2, HSP90B1, JUN, MAPK3, UBE2S, VEGFA	–1.539
mmu-miR-204_st	MMP9	–1.42
mmu-miR-205_st	PTEN, VEGFA	**–12.725**
mmu-miR-214_st	ATF4	–2.004
mmu-miR-214_st	PTEN	–2.004
mmu-miR-217_st	IRS1, PTEN	**–3.008**
mmu-miR-23a_st	PTEN	–1.492
mmu-miR-26b_st	PTEN	–1.675
mmu-miR-27b_st	GRB2, MMP13	–2.025
mmu-miR-29a_st	PIK3R1, PTEN	–2.403
mmu-miR-30a-star_st	PIK3C2A	–2.963
mmu-miR-30c_st	JUN, TP53, UBE2I	–1.54
mmu-miR-31_st	**HIF1A**	**2.492**
mmu-miR-335-5p_st	PTPN11	1.997
mmu-miR-449a_st	CREB1, TP53, VEGFA	–1.416
mmu-miR-3107_st	PTEN	–1.461
mmu-miR-494_st	PTEN	1.377
mmu-miR-504_st	VEGFA	–1.445
mmu-miR-25_st	PTEN	–1.394

**Table 6:** Pathway analysis 3

miRNA (HET)	Target genes	Fold change
**miRNAs related to Autophagy**		
mmu-let-7f_st	VPS39	–1.552
mmu-miR-206_st	BCL2	**–4.864**
mmu-miR-206_st	CTSC	**–4.864**
mmu-miR-100_st	MTOR	–1.78
mmu-miR-133a_st	RB1CC1	**–3.717**
mmu-miR-143_st	BCL2	–1.073
mmu-miR-145_st	LAMP2	–1.137
mmu-miR-153_st	BCL2	–1.179
mmu-miR-155_st	ATG3	–1.133
mmu-miR-195_st	ATG9A	–1.539
mmu-miR-195_st	BCL2	–1.539
mmu-miR-195_st	SQSTM1	–1.539
mmu-miR-106b_st	BCL2	–1.212
mmu-miR-181b_st	BCL2	–1.207
mmu-miR-199a-3p_st	MTOR	**–3.425**
mmu-miR-204_st	CTSC	–1.42
mmu-miR-218_st	CTSB	–1.152
mmu-miR-296-5p_st	BCL2	–1.172
mmu-miR-30c_st	BECN1	–1.54
mmu-miR-449a_st	BCL2	–1.416

**Table 7:** Pathway analysis 4

miRNA (HET)	Target genes	Fold change
**miRNAs related to tight junctions signaling**		
mmu-let-7f_st	TGFBR1	–1.552
mmu-miR-128_st	SNAP25, TGFBR1,	–2.319
mmu-miR-132_st	**TJP1**	–1.818
mmu-miR-133a_st	RHOA	–3.717
mmu-miR-195_st	CLDN12, JUN, NAPG, PPP2R5C, VTI1B	–1.539
mmu-miR-3473_st	AKT1, CDC42, RHOA	3.038
mmu-miR-19b_st	PTEN	1.459
mmu-miR-200c_st	PTEN	–5.193
mmu-miR-205_st	PTEN	–12.725
mmu-miR-214_st	PTEN	–2.004
mmu-miR-217_st	PTEN	–3.008
mmu-miR-23a_st	PTEN	–1.492
mmu-miR-26b_st	PTEN, TGFBR2	–1.675
mmu-miR-29a_st	CDC42, PTEN, TGFB3	–2.403
mmu-miR-30c_st	JUN, NAPG, PTPA	–1.54
mmu-miR-31_st	PPP2R2A	2.492
mmu-miR-3107_st	PTEN	–1.461
mmu-miR-494_st	PTEN	1.377
mmu-miR-690_st	CEBPA	1.831
mmu-miR-9_st	NFKB1	–1.709
mmu-miR-25_st	PTEN	–1.394

**Table 8:** Pathway analysis 5

miRNA (HET)	Target genes	Fold change
**miRNAs related to VEGF signaling**		
mmu-let-7f_st	BCL2L1, HRAS, KRAS, NRAS, Ras	–1.552
mmu-miR-206_st	BCL2	**–4.864**
mmu-miR-100_st	FGFR3	–1.78
mmu-miR-351_st	ELAVL1	–1.499
mmu-miR-126-3p_st	IRS1, PIK3R2, VEGFA	–1.748
mmu-miR-146a_st	TLR9	–1.33
mmu-miR-150_st	VEGFA	–1.652
mmu-miR-195_st	BCL2, FGFR1, GRB2, MAP2K1, MAPK3, RAF1, VEGFA	–1.539
mmu-miR-3473_st	AKT1	**3.038**
mmu-miR-199a-5p_st	HIF1A	–1.792
mmu-miR-200c_st	PLCG1	–5.193
mmu-miR-204_st	SHC1	–1.42
mmu-miR-205_st	VEGFA	**–12.725**
mmu-miR-217_st	IRS1	**–3.008**
mmu-miR-27b_st	FOXO1, GRB2, PXN	–2.025
mmu-miR-29a_st	PIK3R1	–2.403
mmu-miR-30a-star_st	PIK3C2A	–2.963
mmu-miR-31_st	HIF1A	2.492
mmu-miR-335-5p_st	PTPN11, PXN	1.997
mmu-miR-449a_st	BCL2, MAP2K1, VEGFA	–1.416
mmu-miR-3107_st	FOXO1	–1.461
mmu-miR-491_st	BCL2L1	–2.705
mmu-miR-504_st	VEGFA	–1.445
mmu-miR-9_st	FOXO1	–1.709
**miRNAs related to Apoptosis**		
mmu-miR-206_st	BCL2	**–4.864**
mmu-miR-125b-2-3p_st	BAK1, BAX, TP53	–1.495
mmu-miR-351_st	BAK1, CASP6, CASP7, MAP2K7, TP53	–1.499
mmu-miR-132_st	CAPN8	–1.818
mmu-miR-133a_st	CASP9, MCL1	**–3.717**
mmu-miR-146a_st	CHUK	–1.33
mmu-miR-195_st	BCL2, RAF1, MAP2K1, MAP2K4, MAPK3, MCL1	–1.539
mmu-miR-19b_st	BCL2L11	1.459
mmu-miR-200c_st	PLCG1	**–5.193**
mmu-miR-205_st	PRKCE	**–12.725**
mmu-miR-214_st	BAX	–2.004
mmu-miR-26b_st	BAK1	–1.675
mmu-miR-27b_st	BAX	–2.025
mmu-miR-29a_st	MCL1	–2.403
mmu-miR-30c_st	MAP4K4, TP53	–1.54
mmu-miR-449a_st	BCL2, MAP2K1, TP53	–1.416
mmu-miR-382_st	CAPN8	–1.447
mmu-miR-487b_st	MAP2K4	–1.591
mmu-miR-491_st	BCL2L1	–2.705
mmu-miR-9_st	NFKB1	–1.709
mmu-miR-25_st	BCL2L11, MAP2K4	–1.394
**miRNAs related to gap junctions signaling**		
mmu-miR-206_st	EGFR	–4.864
mmu-miR-100_st	FGFR3	–1.78
mmu-miR-126-3p_st	IRS1, PIK3R2	–1.748
mmu-miR-200a_st	CTNNB1	1.157
mmu-miR-195_st	EGFR, FGFR1, GRB2, MAP2K1, MAPK3, RAF1	–1.539
mmu-miR-3473_st	AKT1	3.038
mmu-miR-200c_st	PLCG1	–5.193
mmu-miR-205_st	PRKCE	–12.725
mmu-miR-217_st	IRS1	–3.008
mmu-miR-27b_st	GRB2	–2.025
mmu-miR-29a_st	PIK3R1, SP1, TUBB2A	–2.403
mmu-miR-30a-star_st	PIK3C2A, TUBA1A	–2.963
mmu-miR-30c_st	GNAI2, PPP3CA	–1.54
mmu-miR-335-5p_st	PTPN11	1.997
mmu-miR-449a_st	MAP2K1	–1.416

### HHcy induces dysregulation of miRNAs targeting DNMTs or HDACs

**Figure 6:**
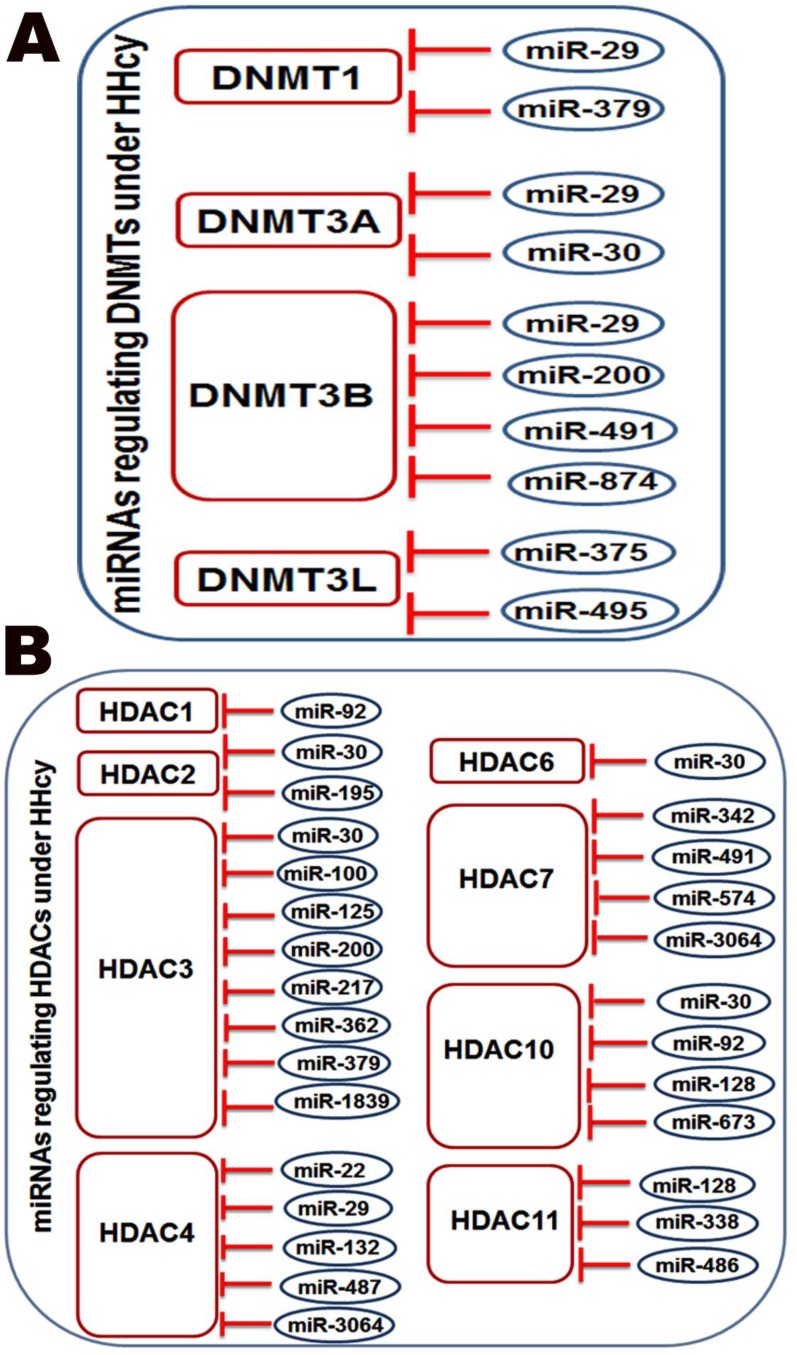
The pathway building tool of IPA showed that miRNAs downregulated by HHcy are targeting enzymes responsible for DNA methylation (DNMT1, DNMT3A, DNMT3B and DNMT3L) as shown in (**A**) and histone deacetylation (HDAC1, HDAC2, HDAC3, HDAC4, HDAC6, HDAC7, HDAC10 and HDAC11) (**B**).

To study the possible HHcy-induced interaction between miRNAs and DNMTs/HDACs enzymes, IPA pathway building tools were used as a powerful tool to predict miRNAs that are targeting different types of DNMTs or HDACs. MiRNA node was used to analyze DNMT1, DNMT2, DNMT3, DNMT4, HDAC1, HDAC2, HDAC3, HDAC4, HDAC5, HDAC6, HDAC7, HDAC8, HDAC9, HDAC10 and HDAC11 molecules. The analysis revealed miRNAs targeting these enzymes (Table 9A–9B). Further analysis identified that HHcy induced downregulation of a number of miRNAs that target DNMTs or HDACs (Figure 6A and 6B). Among the downregulated miRNAs; miR-29 was found to target DNMT1, DNMT3A, DNMT3B and HDAC4),while miR-30 targets DNMT3A, HDAC2, HDAC3, HDAC6 and HDAC10, miR-379 targets DNMT1 and HDAC3 and miR-491 (miR-491 targets DNMT3B and HDAC7.

**Table 9A:** miRNAs targeting DNMTs

DNMTs	miRNAs
**DNMT1**	miR-1278, mir-133, miR-148a-3p, miR-185-5p, miR-21-3p, mir-29, miR-3529-3p, miR-379-5p, miR-3922-5p, miR-4450, miR-4451, miR-450b-3p, miR-4536-5p, miR-4650-3p, miR-4717-5p, miR-5003-5p, miR-548ae-3p, miR-5700, miR-6782-5p, miR-6857-5p, miR-6888-5p, miR-887-3p
**DNMT2**	miR-1226-3p, miR-1246, miR-1252-5p, miR-1290, miR-146a-3p, miR-32-3p, miR-4668-3p, miR-4717-3p, miR-4733-3p, miR-4802-3p, miR-490-5p, miR-5000-5p, miR-5007-3p, miR-5096, miR-513a-3p, miR-591, miR-6817-3p, miR-8080
**DNMT3A**	miR-101-3p, miR-1183, miR-122, miR-1224-3p, miR-1260a, miR-143-3p, miR-181a-1-3p, miR-1900, miR-196b-3p, miR-199, miR-199a-3p, miR-219b-5p, miR-29, miR-29b-3p, miR-29c-5p, miR-3065-3p, miR-3083-5p, miR-30c-5p, miR-3117-3p, miR-3169, miR-3183, miR-3202, miR-331-3p, miR-3547, miR-361, miR-3612, miR-363-5p, miR-381-3p, miR-3911, miR-4256, miR-4290, miR-4300, miR-4307, miR-4314, miR-4485-5p, miR-4525, miR-4687-5p, miR-4694-5p, miR-4704-3p, miR-4708-3p, miR-4716-3p, miR-4723-5p, miR-4740-5p, miR-4747-5p, miR-4768-3p, miR-5010-3p, miR-5196-3p, miR-532-3p, miR-548as-3p, miR-598-3p, miR-6078, miR-6084, miR-670-3p, miR-6741-5p, miR-6772-3p, miR-6776-5p, miR-6780a-3p, miR-6801-3p, miR-6804-3p, miR-6825-5p, miR-6861-5p, miR-6881-3p, miR-6895-3p, miR-6929-5p, miR-6976-5p, miR-7032-3p, miR-7110-3p, miR-718, miR-7515, miR-8068
**DNMT3B**	miR-1180-5p, miR-1204, miR-124-3p, miR-1249-5p, miR-1275, miR-1283, miR-1301-3p, miR-148, miR-148a-3p, miR-185-5p, miR-200b-3p, miR-2277-3p, miR-29, miR-296-5p, miR-299a-3p, miR-29a-5p, miR-29b-3p, miR-3103-5p, miR-3144-5p, miR-3157-5p, miR-324-3p, miR-339-5p, miR-349, miR-3547, miR-3618, miR-3651, miR-3921, miR-4271, miR-4310 miR-432, miR-4323, miR-4434, miR-4440, miR-4472, miR-4525, miR-4660, miR-4668-5p, miR-4676-5p, miR-4682, miR-4694-5p, miR-4758-3p,miR-4771, miR-4800-3p, miR-491-5p, miR-5129-5p, miR-5192, miR-548ag, miR-550a-5p, miR-5584-5p, miR-5682, miR-5684, miR-6132, miR-625-5p, miR-6716-5p, miR-6724-5p, miR-6732-5p, miR-6734-5p, miR-6742-3p, miR-6742-5p, miR-6747-5p, miR-6752-5p, miR-6755-5p, miR-6777-5p, miR-6801-3p, miR-6811-3p, miR-6834-5p, miR-6839-3p, miR-6851-3p, miR-6852-5p, miR-6867-3p, miR-6873-5p, miR-7976, miR-874-3p, miR-885-3p, miR-938, miR-939-3p
**DNMT3L**	miR-1264, miR-3551-5p, miR-3589 miR-375-3p miR-3915, miR-3928-3p, miR-4423-5p, miR-4677-5p, miR-495-5p, miR-6124, miR-6739-3p, miR-6837-3p

**Table 9B:** miRNAs targeting HDACs

HDACs	miRNAs
**HDAC1**	miR-1, miR-1185-5p, miR-1188-3p, miR-1304-5p, miR-146, miR-188-5p, miR-1914-3p, miR-2110, miR-214-3p, miR-218-2-3p, miR-3121-3p, miR-3126-5p, miR-3127-3p, miR-3150a-3p, miR-3200-5p, miR-34a-5p, miR-3614-5p, miR-3616-3p, miR-3919, miR-423-5p, miR-4266, miR-4269, miR 4271, miR-4284, miR-4292, miR-4293, miR-4433a-3p, miR-4434, miR-4436b-3p, miR-4450, miR-450b-3p, miR-4514, miR-4667-5p, miR-4672, miR-4681, miR-4690-5p, miR-4747-5p, miR-4768-3p, miR-4786-3p miR-500b-3p,miR-5090, miR-512-5p, miR-520d-5p, miR-5680, miR-584-5p, miR-593-5p, miR-6165, miR-671-5p, miR-6721-5p, miR-6727-5p, miR-6734-5p, miR-6768-5p, miR-6809-5p, miR-6810-5p, miR-6815-5p, miR-6834-5p, miR-6852-5p, miR-6857-5p, miR-6888-5p, miR-6894-5p, miR-758-5p, miR-7976, miR-8075, miR-889-5p, miR-92a-1-5p
**HDAC2**	mir-145, miR-155,miR-1668, miR-192-3p, miR-195-3p, miR-3087-3p, miR-30a-3p, miR-3165, miR-3682-5p, miR-383-5p, miR-3977, miR-4275, miR-4328, miR-4422, miR-4434, miR-4480, miR-4531, miR-455-3p, miR-4753-5p, miR-4786-3p, miR-548ad-3p, miR-5581-3p, miR-580-5p, miR-6872-3p, miR-6880-5p, miR-6885-3p, miR-7843-3p, miR-8071
**HDAC3**	miR-100-5p, miR-1225-3p, miR-125a-3p, miR-1261, miR-1273h-5p, miR-1298-3p, miR-1302, miR-146a-5p, miR-1839-5p, miR-1910-5p, miR-1915-3p, miR-1972, miR-200b-3p, miR-204-3p, miR-216b-5p, miR-217, miR-30c-1-3p, miR-3127-3p, miR-3162-5p, miR-3189-3p, miR-324-3p, miR-326, miR-328-5p, miR-330-5p, mir-335,miR-3584-5p, miR-3594-5p, miR-362-5p, miR-384,miR-3913-5p, miR-3934-5p, miR-3936,miR-4264, miR-4292, miR-4308, miR-4314, miR-4433b-5p, miR-4446-5p, miR-452-3p, miR-455-3p, miR-4650-5p, miR-4688, miR-4773, miR-483-3p, miR-504-3p, miR-505-5p, miR-589-3p, miR-589-5p, miR-619-5p, miR-6510-3p, miR-6510-5p, miR-6512-3p, miR-6515-3p, miR-6729-3p, miR-6749-3p,miR-6758-5p, miR-6769a-3p, miR-6809-5p, miR-6825-5p, miR-6827-5p, miR-6828-5p, miR-6834-3p, miR-6846-5p, miR-6859-3p, miR-6861-5p, miR-6870-3p, miR-6873-3p, miR-6878-5p, miR-6929-5p, miR-6967-5p, miR-6976-5p, miR-7064-3p, miR-7110-3p, miR-766-3p, miR-766-5p, mir-8
**HDAC4**	mir-1,miR-1-3p, mir-10,miR-1181, miR-1225-3p, miR-124-3p, miR-132-5p, mir-140,miR-140-5p, miR-187-3p, miR-1908-3p, miR-1913, mir-22,miR-29b-3p, mir-302,miR-3064-3p, miR-3072, miR-3144-3p, miR-3186-5p, miR-370-3p, miR-3943, miR-4258, miR-4449, miR-4462, miR-4681, miR-487b-3p, miR-5008-3p, miR-602, miR-6717-5p, miR-6759-5p, miR-6774-3p, miR-6814-3p, miR-6820-5p, miR-6889-3p, miR-7108-3p, miR-7161-3p, miR-8,miR-8068, miR-9-3p, miR-92b-5p, miR-937-3p
**HDAC5**	miR-124-3p,miR-2861, miR-3118, miR-3164, miR-6818-5p, miR-9-3p
**HDAC6**	miR-1181, miR-1207-3p, miR-1227-5p, miR-1285-3p, miR-1915-5p, miR-2392, miR-3072, miR-30b-3p,miR-3150a-3p,miR-3175, miR-3200-3p, miR-337-3p, miR-3473b, miR-3677-3p, miR-378g, miR-4258, miR-433-3p, miR-4438, miR-4659a-5p, miR-4684-3p, miR-4690-5p, miR-4723-5p, miR-4749-3p, miR-4763-5p, miR-4764-3p, miR-506-5p, miR-5095, miR-5135, miR-518a-5p, miR-5195-5p, miR-6131, miR-6721-5p, miR-6762-5p, miR-6872-3p, miR-6887-3p, miR-762
**HDAC7**	miR-1228-5p,miR-1237-5p, miR-1249-5p, miR-1275, miR-140-5p, miR-1896, miR-1900, miR-1915-3p, miR-218-1-3p, miR-3064-5p, miR-3109-3p, miR-3150a-3p, miR-3157-5p, miR-3175, miR-3191-5p, miR-328-5p, miR-342-3p, miR-3594-5p, miR-3649, miR-377-3p, miR-4292, miR-4472, miR-4489, miR-4512, miR-4638-5p, miR-4651, miR-4690-5p, miR-4691-5p, miR-4723-5p, miR-4788, miR-491-5p, miR-5006-5p, miR-5129-5p, miR-516a-5p, miR-5193, miR-520a-5p, miR-541-3p, miR-544b, miR-5689, miR-574-3p, miR-593-5p, miR-615-5p, miR-637, miR-6515-5p, miR-660-3p, miR-669c-3p, miR-6742-5p, miR-6756-5p, miR-6764-3p, miR-6768-5p, miR-6825-5p, miR-6846-5p, miR-6859-3p, miR-6861-3p, miR-6862-5p, miR-6875-3p, miR-6887-3p, miR-7112-5p, miR-7113-5p, miR-7155-5p, miR-7974, mir-8,miR-92a-2-5p, miR-96-5p
**HDAC8**	let-7c-1-3p, miR-101-3p, miR-10a-5p, miR-1179, miR-1260a, miR-1273g-3p, miR-1278, miR-1295b-5p, miR-1301-5p, miR-139-5p, miR-144-3p, miR-146a-5p, miR-150-3p, miR-150-5p,miR-152-5p, miR-16-1-3p, miR-181a-5p, miR-182-3p, miR-188-3p, miR-194-5p, miR-1953, miR-197-5p, miR-202-5p, miR-203a-5p, miR-2110, miR-2115-5p, miR-216b-3p, miR-216b-5p, miR-218-2-3p, miR-26b-3p, miR-3058-5p, miR-3090-3p, miR-31-3p, miR-3151-3p, miR-3611, miR-3659, miR-3675-3p, miR-376c-3p, miR-377-3p, miR-4267, miR-4271, miR-4274, miR-4279,miR-4291, miR-4307, miR-4461, miR-4487, miR-450a-1-3p, miR-4645-5p, miR-4650-3p, miR-4666a-5p, miR-466d-5p, miR-4686, miR-4711-5p, miR-4716-3p, miR-4718, miR-4723-5p, miR-4760-3p, miR-4768-5p, miR-4769-3p, miR-4777-3p, miR-488-5p, miR-497-3p, miR-504-5p, miR-506-5p,miR-5089-3p, miR-509-5p, miR-5112, miR-5193, miR-5579-5p, miR-5581-3p, miR-5683, miR-5693, miR-5696, miR-584-5p, miR-6501-5p, miR-6509-3p, miR-6512-3p, miR-655-5p, miR-664-3p, miR-668-3p, miR-6734-3p, miR-6755-5p, miR-6758-5p, miR-6806-5p, miR-6818-5p, miR-6825-5p, miR-6844, miR-6873-3p, miR-6883-3p, miR-6895-3p, miR-7154-3p, miR-7159-3p, miR-743a-5p, miR-744-3p, miR-7a-5p, miR-891b
**HDAC10**	miR-1199-3p,miR-1228-5p, miR-1233-5p, miR-1237-5p, miR-1273h-5p, miR-128-1-5p, miR-1282, miR-1293, miR-1294, miR-1343-5p, miR-1893, miR-1908-3p, miR-1908-5p, miR-1914-3p, miR-3080-5p, miR-30c-1-3p,miR-3141, miR-3178, miR-3180-3p, miR-337-5p, miR-363-5p, miR-3648, miR-381-5p, miR-423-5p, miR-4481, miR-4634, miR-4649-5p, miR-4651, miR-4706, miR-4710, miR-4734, miR-4743-5p, miR-4746-3p, miR-4781-5p, miR-504-3p, miR-542-5p, miR-5707, miR-5708, miR-6090,miR-6134, miR-615-5p, miR-673-3p, miR-6734-5p, miR-6756-5p, miR-6777-5p, miR-6781-5p, miR-6782-5p, miR-6827-5p, miR-6853-5p, miR-6880-5p, miR-744-5p, miR-7704, miR-7854-3p, miR-8055, miR-92b-5p
**HDAC11**	miR-1207-5p, miR-1224-3pmiR-1225-3p, miR-1227-5p, miR-1247-5p, miR-1254, miR-1258, miR-1260a, miR-128-1-5p, miR-1291, miR-1343-3p, miR-1587, miR-1825, miR-1843a-5p, miR-18a-3p, miR-1909-3p, miR-1913, miR-1934-5p, miR-1972, miR-214-5p, miR-2392, miR-23a-5p,miR-296-5p, miR-3070-5p, miR-3072, miR-3084-3p, miR-3103-5p, miR-3104-5p, miR-3109-3p, miR-3110-5p, miR-3127-3p, miR-3152-5p, miR-3162-5p, miR-3180-3p, miR-3200-3p, miR-324-5p, miR-331-3p, miR-338-3p, miR-346, miR-361-3p, miR-3616-3p, miR-3649, miR-3689d, miR-3909, miR-3918, miR-4254, miR-4290, miR-4292, miR-4308, miR-4446-3p, miR-4455, miR-4512, miR-4527, miR-4530, miR-4534, miR-4640-5p, miR-4650-5p, miR-4656, miR-4695-5p, miR-4716-3p, miR-4717-5p, miR-4747-3p, miR-4749-3p, miR-4763-5p, miR-486-3p, miR-486-5p, miR-492, miR-5008-5p, miR-5088-3p, miR-5195-5p, miR-550a-5p, miR-6081, miR-6132, miR-639, miR-6508-3p, miR-6510-5p, miR-6511b-5p, miR-655-5p, miR-659-5p, miR-661, miR-6734-5p, miR-6735-3p, miR-6747-3p, miR-6748-3p, miR-6749-3p, miR-6752-3p, miR-6762-5p, miR-6778-3p, miR-6791-3p, miR-6801-3p, miR-6802-3p, miR-6803-3p,miR-6808-5p, miR-6825-5p, miR-6836-3p, miR-6841-3p, miR-6862-5p, miR-6882-3p, miR-6887-3p, miR-6892-3p, miR-6967-5p, miR-7032-3p, miR-7064-3p, miR-7108-3p, miR-7113-3p, miR-762, miR-766-3p, miR-7705, miR-7851-3p, miR-939-3p

## DISCUSSION

Recently, we reported deleterious effects of elevated Hcy on BRB integrity [2, 4, 76]. Elevated Hcy level was reported as an epigenetic modulator in various diseases such as chronic kidney disease [77], cardiovascular diseases [78] and atherosclerosis [79]. In the present study, epigenetic modifications were proposed as possible mechanisms of HHcy-induced BRB dysfunction. Currently, it is believed that this is the first study to screen for the epigenetic changes that occur in retina and retinal endothelial cells under HHcy. The major findings of the current study are 1) Hcy induced a significant increase of both HDACs and DNMTs activity in mouse retina, cultured HRECs and RPE; 2) Inhibition of DNMT and HDAC ameliorates Hcy-induced BRB dysfunction. 3) miRNA profiling of *cbs^+/–^* and *cbs^–/–^* detected 127 miRNAs in *cbs^+/–^* that were statistically significant and differentially expressed in comparison to *cbs^+/+^.*4) We detected similarity between some miRNAs altered under HHcy and miRNAs altered in DR or AMD. 4) miRNA pathway analysis showed HHcy-dysregulated miRNAs involvement in ER stress, oxidative stress, inflammation, angiogenesis, and hypoxia pathways. 5) DNMTs and HDACs are direct targets of miRNAs dysregulated in HHcy.

Among different epigenetic pathways, DNA methylation is the major epigenetic modification associated with HHcy. Methylation of DNA is mediated by DNMTs and requires the presence of S-adenosylmethionine (SAM), a methyl donating compound that delivers methyl groups to maintain other metabolic reactions in human body [80]. Hcy is an intermediate product released during methionine cycle. Methionine combines with adenosine to produce SAM. After releasing its methyl group, SAM is converted to SAH which is further hydrolyzed to Hcy and adenosine [81]. Therefore, the Hcy-methionine cycle is very critical for maintenance of a balanced amount of SAM, and therefore normal DNMTs activity. Indeed, differential levels of DNA methylation, from hyper- to hypo-methylation, have been reported to be induced by HHcy [77]. A likely explanation for such phenomena is the fact that other mechanisms are involved in regulating DNA methylation and histone modification. In the current study, the Hcy increased DNMTs activity both *in vivo* and *in vitro*, suggesting HHcy-induced global increase in retinal DNA methylation.

DNA methylation has been reported to cooperate with histone deacetylation to mediate transcriptional repression [82]. Furthermore, increased level of HDAC was reported in many ocular diseases such as ischemic retinal injury [83], retinal degenerative diseases [84], optic nerve injury [85] and corneal neovascularization [86]. In addition, increased histone deacetylase (HDAC) activity with subsequent dysregulation of protein acetylation has been linked to retinal degenerations associated with ischemia and ocular hypertension [87]. Our results showed increased HDAC activity both *in vivo* and *in vitro* (mice models of HHcy, HRECs and ARPE-19 cells treated with Hcy).

miRNAs play a crucial role in regulation of gene expression and their altered expression has been reported in serum of patients with various eye diseases including DR [51–55] and AMD [56–59], and also in cardiovascular diseases [88, 89]. The current study evaluated the role of miRNAs in HHcy-induced BRB dysfunction and related these miRNAs changes to retinal diseases associated with BRB dysfunction such as DR and AMD. MiRNAs expression were analyzed and yielded differential expression in HHcy mice retina (*cbs^+/–^* and *cbs^–/–^*) compared to *cbs^+/+^* control.

Interestingly, epigenetic modifications were reported to play a crucial role in HHcy induced-blood brain barrier dysfunction, with profound involvement of miRNAs in the pathogenesis of the leaky cerebral vasculature in *cbs^+/–^* mice [90].

To narrow the focus, the differentially expressed miRNAs in both *cbs^+/–^* and *cbs^–/–^* mice were compared to identify commonly expressed miRNAs in HHcy. The analysis revealed 25 miRNAs were similarly affected by elevated Hcy in both models. Furthermore, miRNAs were reported to be linked to the pathogenesis of diabetic complications [91]. Therefore, we evaluated that the differentially expressed miRNAs in mouse models of elevated Hcy and in STZ-mice as a model of Type 1 diabetes. The analysis revealed 6 miRNAs commonly differentially expressed in both STZ and HHcy mice. Among those miRNAs, 2 miRNAs (miR-16-5p and miR-194) were consistently changing among the three different groups. Interestingly, miR-16-5p has been reported to be tissue protective and to be decreased in a diabetic rat's kidney [92].

In addition to the reported role of HHcy in DR [15, 93, 94], our previous work reported that increased Hcy was associated with features of AMD in mice including RPE dysfunction [4]. Therefore, the current study also aimed to evaluate the similarity of miRNAs profile in mouse models of HHcy and what has been reported in patient with AMD, especially various miRNAs were linked to AMD pathogenesis [95]. The study revealed downregulation of miR-205, miR-27, miR-31, and miR-29 in the *cbs^+/–^* retinas, these miRNAs were also reported to be downregulated in vitreous [68] and plasma of AMD patients [69]. Furthermore, miR-31 which was altered in *cbs^+/–^* retinas was suggested as a regulator of choroidal neovascularization [70]. The analysis revealed similarity in miRNAs differentially expressed in both AMD patients and retina of *cbs^+/–^* mice.

Recently, studies uncovered evidence that miRNAs travel around the body are contained within exosomes [96]. Increasing evidence points to exosomes and micro vesicles as a unique alternative mechanism for protein transfer and intercellular communication, allowing exchange of proteins, lipids and nucleic acids between cells [97]. Accumulating evidence supported the role of exosomal miRNAs in eye diseases [98, 99].

Related to the fact that there is no definite animal model of AMD and numerous models mimic some of the significant pathological features seen in AMD, however none recreated all of its characteristics. That is why, having an animal model that mimics both the early and late features of AMD has been challenging [100]. Our previous work reported that, HHcy induces features of AMD both *in vivo (cbs mice)* and *in vitro* (Hcy-treated RPE) [4]. Consequently, we evaluated miRNA differential profile in exosomes released from Hcy-treated RPE. Two miRNAs (MiR-17-5p and let-7a-3p) were found to be commonly differentially expressed in retinas of *cbs^–/–^* mice, diabetic mice as well as RPE exosomes. Interestingly, those two miRNAs were also reported to be altered in DR and AMD [91, 101, 102]. Moreover, miR-17-5p has been reported to be involved in both pro- and anti-proliferative signals via dysregulation of normal cell cycle [103].

In conclusion, understanding HHcy-induced epigenetic modifications lead to possible prospects in evaluating miRNAs as molecular biomarkers, prognostic tools, diagnostic tools and therapeutic agents for eye diseases associated with elevated Hcy such as DR and AMD.

## SUPPLEMENTARY MATERIALS FIGURE


