# Supplementary material for: Epigenetic modifications in hyperhomocysteinemia: potential role in diabetic retinopathy and age-related macular degeneration
Source: Oncotarget. 2018 Jan 29;9(16):12562–90. doi: 10.18632/oncotarget.24333 (PMC5849155; doi:10.18632/oncotarget.24333)
Supplement: Supplementary file 1 [file oncotarget-09-12562-s001.pdf]

# Epigenetic modifications in hyperhomocysteinemia: potential role in diabetic retinopathy and age-related macular degeneration

## SUPPLEMENTARY MATERIALS

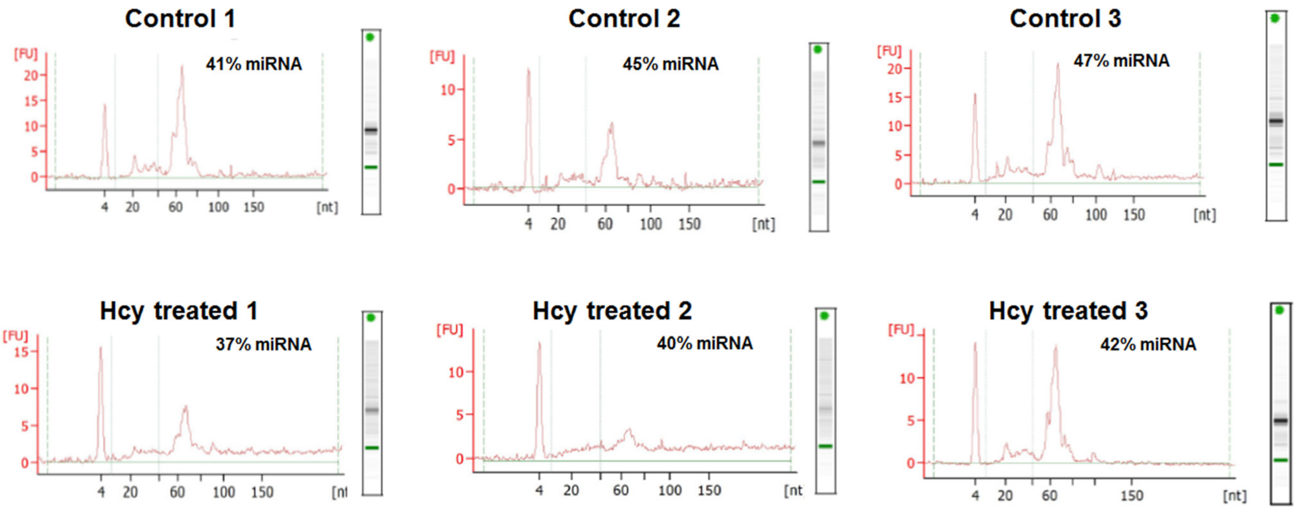


**Supplementary Figure 1: Bioanalyzer data showing concentrations of miRNAs isolated from APRE-derived exosomes.**
